# Multitargeting Histamine H_3_ Receptor Ligands among Acetyl- and Propionyl-Phenoxyalkyl Derivatives

**DOI:** 10.3390/molecules28052349

**Published:** 2023-03-03

**Authors:** Dorota Łażewska, Maria Kaleta, Paula Zaręba, Justyna Godyń, Mariam Dubiel, Ewelina Honkisz-Orzechowska, Agata Doroz-Płonka, Anna Więckowska, Holger Stark, Katarzyna Kieć-Kononowicz

**Affiliations:** 1 Department of Technology and Biotechnology of Drugs, Faculty of Pharmacy, Jagiellonian University Medical College in Kraków, Medyczna 9, 30-688 Kraków, Poland; 2 Department of Physicochemical Drug Analysis, Jagiellonian University Medical College in Kraków, Medyczna 9, 30-688 Kraków, Poland; 3 Institute of Pharmaceutical and Medicinal Chemistry, Heinrich Heine University Düsseldorf, Universitaetsstr. 1, 40225 Düuesseldorf, Germany

**Keywords:** histamine H_3_ receptor ligand, cholinesterase inhibitor, kinetic studies, toxicity, HepG2 lines, SH-SY5Y lines

## Abstract

Alzheimer’s disease (AD) is a neurodegenerative disorder, for which there is no effective cure. Current drugs only slow down the course of the disease, and, therefore, there is an urgent need to find effective therapies that not only treat, but also prevent it. Acetylcholinesterase inhibitors (AChEIs), among others, have been used for years to treat AD. Histamine H_3_ receptors (H_3_Rs) antagonists/inverse agonists are indicated for CNS diseases. Combining AChEIs with H_3_R antagonism in one structure could bring a beneficial therapeutic effect. The aim of this study was to find new multitargetting ligands. Thus, continuing our previous research, acetyl- and propionyl-phenoxy-pentyl(-hexyl) derivatives were designed. These compounds were tested for their affinity to human H_3_Rs, as well as their ability to inhibit cholinesterases (acetyl- and butyrylcholinesterases) and, additionally, human monoamine oxidase B (MAO B). Furthermore, for the selected active compounds, their toxicity towards HepG2 or SH-SY5Y cells was evaluated. The results showed that compounds **16** (1-(4-((5-(azepan-1-yl)pentyl)oxy)phenyl)propan-1-one) and **17** (1-(4-((6-(azepan-1-yl)hexyl)oxy)phenyl)propan-1-one) are the most promising, with a high affinity for human H_3_Rs (*K_i_*: 30 nM and 42 nM, respectively), a good ability to inhibit cholinesterases (**16**: AChE IC_50_ = 3.60 µM, BuChE IC_50_ = 0.55 µM; **17**: AChE IC_50_ = 1.06 µM, BuChE IC_50_ = 2.86 µM), and lack of cell toxicity up to 50 µM.

## 1. Introduction

The pathological cause of Alzheimer’s disease (AD) is neuronal death, which leads to the disruption of connections between neurons and the inhibition of the processing and conduction of electrical impulses. This is caused by the accumulation of protein deposits in neuronal tissue: ß-amyloid or tau protein [1,2]. Recently, it has been indicated that α-synuclein may also play an important role in the pathogenesis of AD [3]. Presumably, there is a feedback loop between all these proteins. β-Amyloid increases GSK-β kinase levels, which induces tau protein phosphorylation and stimulates α-synuclein production. All of this can lead to the aggregation of both the β-amyloid and the tau protein. However, as AD is a disease with a complex a etiology, in addition to the factors discussed above, many more play a role in its development and course [1]. Among these, impaired cholinergic transmission is discussed as an important contributor.

The theory of the involvement of acetylcholine (ACh) in the development of AD has been known since the 1970s [4], and is based on the observation of reduced levels of this neurotransmitter in the brains of AD patients, leading to problems with cognition and memory. Cholinesterases (acetyl- and butyrylcholinesterase), especially acetylcholinesterase (AChE), play important roles in the degradation of ACh, which, under normal conditions, ensures the proper transmission of neuronal signals. Under pathological conditions, when these enzymes are overexpressed, ACh can be over-degraded, and many factors important in the development of AD may be affected (e.g., β-amyloid formation, tau protein phosphorylation, or neuroinflammation) [5]. One possible way to increase ACh levels is to reduce the activity of these enzymes by using their inhibitors. In the 1990s, the first AChE inhibitors were introduced to therapy (tacrine-1993 and donepezil-1996), followed by others in the following years (rivastigmine-2000 and galantamine 2001), and are used (except tacrine, which was withdrawn in 2013) as key drugs in the treatment of AD [6].

Since then, the search for other active AChE inhibitors has been ongoing, and many compounds based on the structures of known inhibitors or structurally new compounds have been described in the literature [7]. Recently, the interest has focused on the search for multitarget ligands, as it is believed that such compounds may be more effective in the treatment of diseases with multiple etiologies, including AD [8]. Among these compounds, ligands are described that act simultaneously on AChE and histamine H_3_ receptors (H_3_R) [9]. H_3_Rs were first described in the 1980s by Arrang et al. [10]. They are mainly located presynaptically in neurons of the central nervous system (CNS), especially in regions associated with memory, cognition, and arousal [11,12]. H_3_Rs influence the level of histamine itself and affect the release of other neurotransmitters, including ACh. The blocking of H_3_Rs increases neurotransmitter release. Many preclinical studies suggest the utility of H_3_R antagonists/inverse agonists in different CNS diseases, including AD [13,14]. Although clinical studies with H_3_R ligands did not show higher benefits than the currently used AChE inhibitors, it is believed that multitarget compounds combining these two activities may have more positive effects [15]. Quite recently, a crystal structure of human H_3_R (hH_3_R) in a complex with PF-03654746 (H_3_R ligand) was described [16] showing the conservative mode of ligand interactions and hydrophobic contacts in the bottom part of the binding pocket. This finding may be very helpful in the search for new potent compounds.

**Figure 1:**
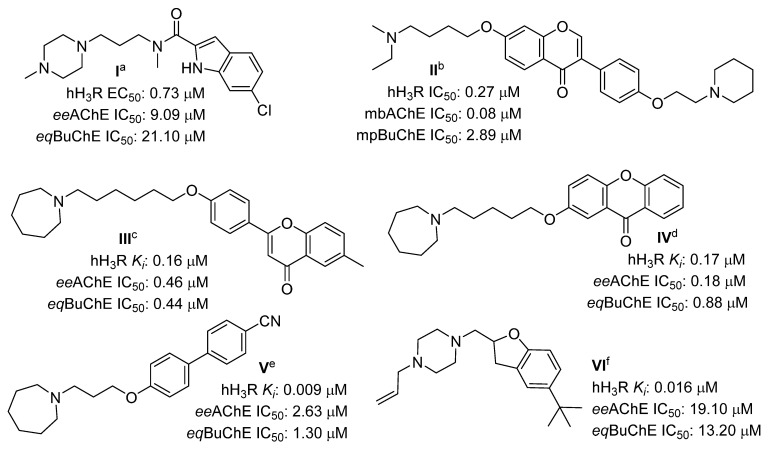
Structures of the most promising histamine H_3_R ligands with cholinesterase inhibitory activity from the last 3 years. hH_3_R—human H_3_R; *ee*AChE—AChE from electric eel; *eq*BuChE—BuChE from equine serum; mbAChE—AChE extracted from mouse brains; mpAChE—AChE extracted from mouse plasma. ^a^ Data from [17]; ^b^ data from [18]; ^c^ data from [19]; ^d^ data from [20]; ^e^ data from [21]; ^f^ data from [22].

In recent years, many interesting papers have been published, describing multitarget H_3_R ligands with potential use in AD therapy [17,18,19,20,21,22]. The structures of the most promising H_3_R compounds with cholinesterase inhibition activities in the last three years are shown in Figure 1. Some of them, such as compounds **IV** and **V** (Figure 1), showed inhibitory activity targeting monoamine oxidase B (MAO B). MAO B inhibitors are known to increase the level of dopamine (DA) in the brain, stopping its metabolism and reducing reactive oxygen species produced during this metabolism [23]. Moreover, the latest studies show that DA levels are reduced in AD patients [24]. Furthermore, it is thought that DA may also play an important role in the pathogenesis of AD [25].

The aim of this study was to synthesize and pharmacologically test acetyl- and propionylphenoxyalkyl derivatives designed, on the basis of our previous work, as H_3_R ligands and cholinesterase inhibitors [20,26]. The compounds obtained were tested in in vitro assays to estimate their pharmacological activity (H_3_Rs, AChE, BuChE, MAO B). Moreover, selected compounds were evaluated for any toxic effects on HepG2 and SH-SY5Y cell lines.

## 2. Results

### 2.1. Design of Compounds

**Figure 2:**
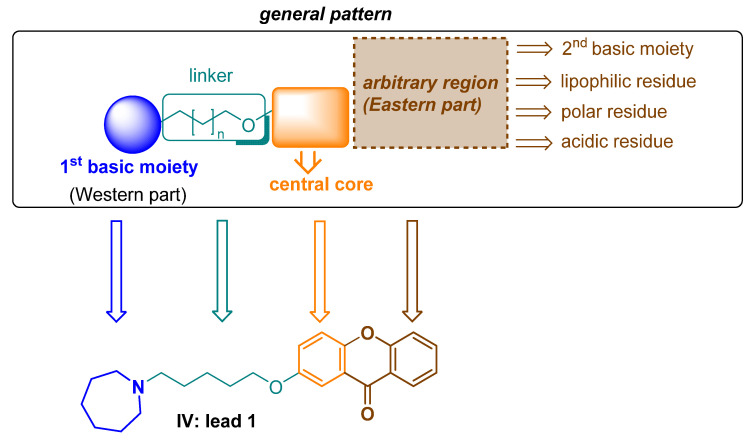
Pharmacophore model of H_3_R antagonists/inverse agonists and the **lead 1**.

Three years ago, through in silico studies (molecular modeling and docking to cholinesterases), xanthones were found to be potential ligands for H_3_R, cholinesterases and MAO B [20]. The designed compounds aligned with the general construction pattern of previously suggested H_3_R antagonists/inverse agonists (Figure 2) [27].

**Figure 3:**
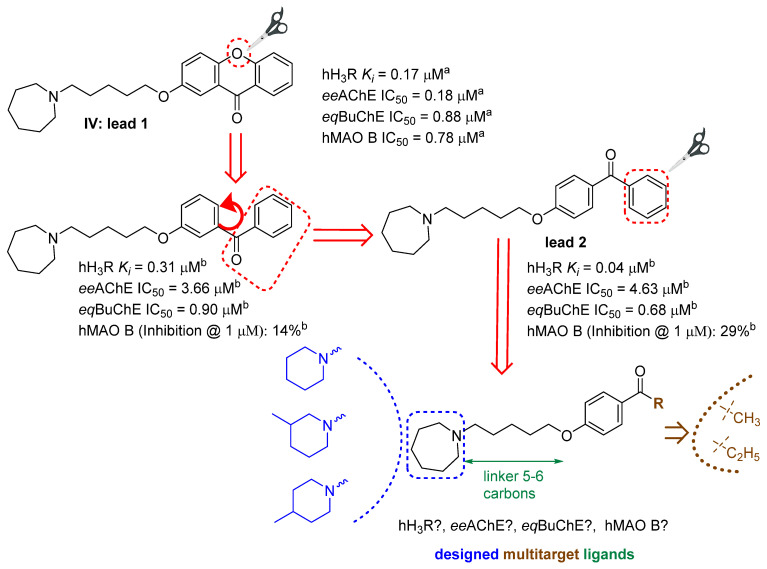
Lead compounds and modification strategies. ^a^ Data from [20]; ^b^ data from [26].

Then, xanthones were synthesized, and tested in vitro, in appropriate assays that confirmed their biological activity against these targets, which have been previously described by Łażewska et al. [20]. The most promising structure found in that work, compound **IV** (Figure 1), was selected as the **lead 1** for further modifications, in order to check which fragments of the xanthone moiety affect their pharmacological activity. As a first step, oxygen was removed from this molecule to obtain benzophenone derivatives that have recently been described [26]. The direction of the modifications is shown in Figure 3. Among the benzophenone derivatives, there were structures that showed good activity as multitarget ligands (e.g., the structures in Figure 3). The aim of the current work was to test whether further structural modifications (reduction of substituent size), i.e., replacing the phenyl moiety with an ethyl or a methyl group, would improve or worsen interactions with selected biological targets. In the search for CNS-active compounds (but not exclusively), it is important that compounds have moderate lipophilicity (log P in the range of one to three), as this favorably affects ADMET parameters and increases the compound’s chances of being a drug [28]. Replacing the phenyl ring with an alkyl (methyl, ethyl) substituent provides a reduction in the lipophilicity of the compounds, which was confirmed by preliminary calculations performed using the SwissADME server [29] (data in Supplementary Materials).

### 2.2. Synthesis of Compounds **2**–**17**

**Scheme 1:**
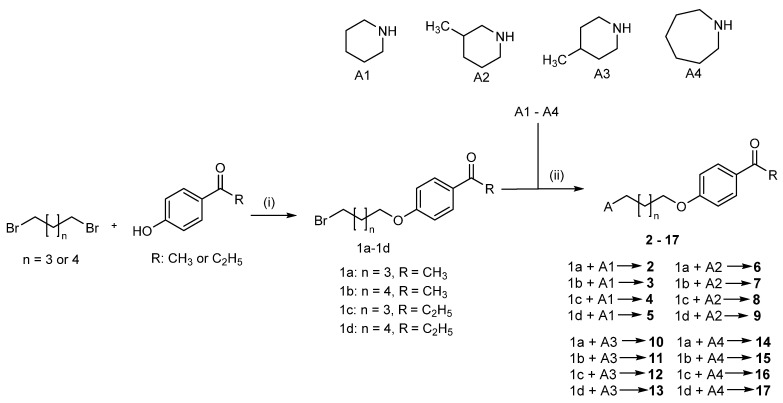
Synthesis of the designed compounds **2**–**17**. Reagents and conditions: (i) Na, 1-propanol, 3 h—60 °C, 3 h—reflux; (ii) K_2_CO_3_, KI, ethanol–water (volume ratio 21:4). Yield: 28–48%.

In the first stage, it was necessary to obtain phenoxyalkylbromides (**1a**–**1d**; Scheme 1) by *O*-alkylation of the corresponding phenols with 1,5-dibromopentane or 1,6-dibromohexane. The reactions were performed in freshly prepared sodium propanolate. The resulting bromides were then subjected to *N*-alkylation with the corresponding amines (piperidines or azepane; Scheme 1). The reactions were carried out in the mixture of ethanol and water (21:4), in the presence of potassium carbonate and catalytic amounts of potassium iodide. The final products were purified by extraction. The oily free bases were transformed into oxalic acid salts. The purity and identity of the products were confirmed by spectroscopic methods (^1^H and ^13^C NMR, LC-MS) and elemental analysis. Spectra data are presented in the Supplementary Materials.

### 2.3. Human Histamine H_3_ Receptor Affinity

The affinity for hH_3_R was assessed in vitro in radioligand binding assays, using membrane preparations of HEK293 cells stably expressing hH_3_R, while [^3^H]-*N*^α^-methylhistamine was used as a radioligand. The exact procedure was described previously by Kottke et al. [30]. Novel compounds were tested as oxalic acid salts. All compounds (except for **9** and **10**) showed very good affinities for these receptors, with *K_i_* values below 100 nM (Table 1). The activity depended on all three variables, i.e., the presence of an amine group, the chain length (five or six carbons), and the type of substituent in the phenyl ring. With regard to the change in the amine moiety, it can be observed that, regardless of the length of the chain and the type of R substituent, the activity of the derivatives was arranged in the following order: 3-methylpiperidine ≥ piperidine ≥ azepane >> 4-metylpiperidine derivatives. Regarding the length of the carbon chain, compounds bearing the pentylene chain are characterized by a higher binding affinity to the receptor than hexylene derivatives (exception: **10**). The change of the acyl group to propionyl one resulted in an almost twofold increase in affinity to hH_3_R receptors (exception **4**). The highest affinity for hH_3_R among all compounds was observed in compound **8**, with a *K_i_* of 12 nM. Seven other compounds (**2**, **4**, **5**, **6**, **9**, **16** and **17**) had also very high affinities, with *K_i_* values < 50 nM.

### 2.4. Cholinesterase Inhibitory Activity

All compounds were tested at a concentration of 10 µM in a modified colorimetric method first described by Ellman et al. in the 1960s [19,31], using AChE from *electrophorus electricus* (*ee*AChE) and BuChE from *equine serum* (*eq*BuChE). The compounds (**5**–**8**, **11**–**13**, **16** and **17**) that showed inhibitions of greater than 70% were selected for further studies, in order to obtain IC_50_ values. All results are collected in Table 1. The IC_50_ values are in the low micromolar range (<5 µM). In general, compounds that showed activity against both cholinoesterases inhibited BuChE to a greater extent than AChE (with the exception of **17**). However, we did not observe any difference in inhibition potency. No correlation was observed between inhibitory activity and hH_3_R affinity. The most interesting compounds were among the azepane derivatives. Compound **16** is the most potent BuChE inhibitor in the whole series, with inhibitory activity in the sub-micromolar range (IC_50_ = 0.55 µM). In contrast, compound **17** shows the highest inhibition of AChE in this series, with a potency of one micromole (IC_50_ = 1.06 µM).

#### Kinetic Studies of the Inhibition of *ee*AChE and *eq*BuChE

For the most active compounds, **17** (AChE inhibitor) and **16** (BuChE inhibitor), tests were carried out to determine the type of inhibition exhibited for the appropriate enzymes. The Michaelis–Menten equation was used to calculate the maximum velocity (V_max_) and the Michaelis constant (K_m_).

**Figure 4:**
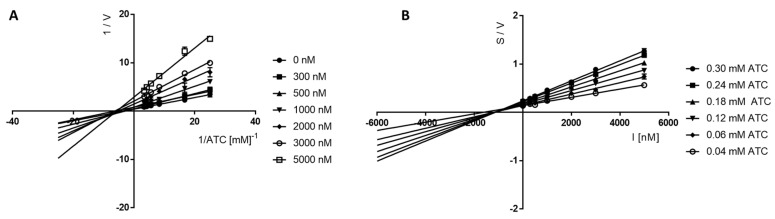
Lineweaver−Burk (**A**) and Cornish−Bowden (**B**) plots illustrating mixed types of *ee*AChE inhibition by compound **17**. S = acetylthiocholine; V = initial velocity rate; I = inhibitor concentration.

Lineweaver–Burk plots obtained for **17** (Figure 4A) showed a series of lines converging at the same point near the x-axis (1/[ATC], which confirmed the non-competitive (mixed) type of *ee*AChE inhibition, as V_max_ decreased with increasing concentrations of inhibitor.

**Figure 5:**
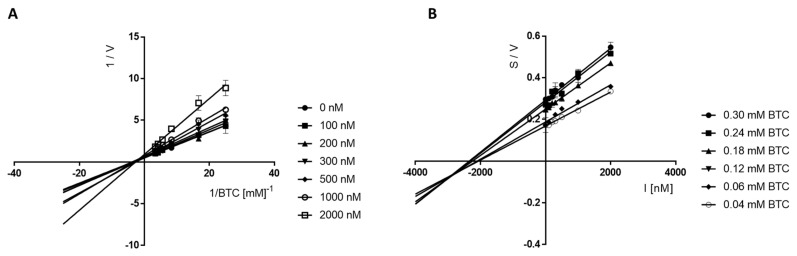
Lineweaver−Burk (**A**) and Cornish−Bowden (**B**) plots illustrating mixed types of *eq*BuChE inhibition by compound **16**. S = butyrylthiocholine; V = initial velocity rate; I = inhibitor concentration.

Furthermore, Cornish–Bowden plots (S/V vs. I) obtained for **17** (Figure 4B) showed a series of lines converging at the same point over the x-axis, thus confirming a mixed mechanism of cholinesterase inhibition. The same situation was observed for compound **16,** for which the type of BuChE inhibition was investigated and evaluated in the Lineweaver–Burk and Cornish–Bowden plots (Figure 5). The type of line intersection and the location of this intersection confirms the mixed type of inhibition.

### 2.5. Monoamine Oxidase B Inhibitory Activity

The ability to inhibit MAO B was estimated by a fluorescence assay, as previously described [32]. The screening assay was carried out at a concentration of 1 µM to determine the percentage inhibition of the enzyme, as compared to reference inhibitors (i.e., pargiline, rasagiline and safinamide). All compounds showed weak percentage of inhibition (<50%) and were not selected for further testing (Table 1).

### 2.6. Cytotoxicity Studies of Selected Compounds

Compounds showing activity against cholinesterases (AChE and/or BuChE) were selected for preliminary evaluation of their toxic effects (i.e., compounds **5**–**8**, **11**–**13**, **16** and **17**) in the MTS assay. The studies were conducted on two cell lines: HepG2 and SH-SY5Y. To assess viability, increasing concentrations of the compounds tested (from 0.78 µM to 50 µM) were incubated with their respective cell lines (HepG2 or SH-SY5Y) for 48 h and 24 h, respectively. After the indicated time, the MTS reagent was added directly to the cultured cells for 1 h, and then the absorbance at 490 nm was read. The study showed that both the tested lines (HepG2 and SH-SY5Y) remained more than 50% viable the highest doses tested. The results are presented in Table 2 and Figures S1 and S2 (Supplementary Materials).

## 3. Discussion

Continuing our previous works on searching for multi-targeted H_3_R ligands, we obtained a series of sixteen compounds designed on the basis of our previous studies [20,26]. We conducted pharmacological tests of acetyl- and propionyl-phenoxy-pentyl (-hexyl) amines, and confirmed the high affinity of these compounds for hH_3_R. The determined *K*_i_ values for all compounds are in the nanomolar range (*K*_i_ < 120 nM). This is in line with our expectations, as these structures fit the proposed pharmacophore model for H_3_R antagonists/reverse agonists [27]. This pharmacophore has the following elements: a basic center, a linker, a central core, and an arbitrary region with high variability, which are required in a molecule for activity. The investigated compounds have all these elements. Among all the used amines (azepane, 3-methylpiperidine, 4-methylpiperidine and piperidine), the least active compounds were the 4-methylpiperidines, and such a correlation was also observed in previous works, e.g., [33]. In regard to the most active compounds, it is difficult to say, without a doubt, as to which amine’s presence is most beneficial. The activities of the derivatives with the other three amines are usually comparable, although compounds with a 3-methylpiperidine moiety are often the most active.

As for the results obtained for cholinesterase inhibition, they are also unclear. No clear correlation was observed between the structure of the compounds and the observed inhibitory potency. Only nine of sixteen compounds showed enough activity in the prescreening (>70% at 10 µM) to qualify them for testing at other concentrations to determine the IC_50_ values. All these compounds showed inhibitory potencies in the low micromolar concentration range (0.55 µM ≤ IC_50_ ≤ 4.79 µM). Some of them inhibited, at micromolar concentrations, both cholinesterases (four compounds); others only inhibited one cholinesterase (five compounds), whereas a low percentage of inhibition (<70%) was observed for seven compounds. These results showed, in comparison with recently described benzophenone derivatives [26], that the exchange of the phenyl substituent for an ethyl substituent and, in particular, a methyl substituent, did not favorably influence cholinesterase inhibition. Almost all of the thirty-four previously described benzophenones (with the exception of three compounds) inhibited the cholinesterases in the micromolar range (0.17 µM ≤ IC_50_ ≤ 7.75 µM) [26], while now only nine compounds showed activity against cholinesterases. Comparing the results for the **lead 2** (Figure 3) directly with analogous compounds **14** (the methyl analogue) and **16** (the ethyl analogue), it was seen that the exchange of the phenyl for the ethyl substituent, in this case, did not affect the activity against all three tested biological targets, i.e., (hH_3_R, AChE and BuChE), very significantly. The opposite result was observed for the methyl substituent (**14**). Shortening the chain to the methyl substituent caused a decrease in activity against both cholinesterases (only 63% of inhibition). Among all nine compounds, compound **16** had the highest BuChE inhibitory activity (IC_50_ = 0.55 µM), whereas compound **17** showed the highest inhibition of AChE (IC_50_ = 1.06 µM). Both these compounds had a hexyl carbon linker.

None of the compounds tested showed a promising inhibition of hMAO B.

All compounds that showed inhibitory activity against cholinesterases were selected for further in vitro studies, in order to evaluate potential toxic effects on HepG2 cells and SH-SY5Y cells in the MTS assay. Human liver carcinoma HepG2 cell lines are the most popular lines to assess the risk of hepatotoxicity [34]. The human neuroblastoma SH-SY5Y lines are used in experimental models of AD to assess intracellular factors that lead to AD (e.g., tau-related pathology [35]) or the neuroprotection ability of ligands. The results of these studies showed that the compounds had very low toxicity. Specific IC_50_ values calculated with non-linear regression, and fitted to a sigmoidal dose–response curve, were higher than 90 µM. The only exception was compound **17,** which exhibited some toxicity against HepG2 cells, with an IC_50_ of 60 µM.

In summary, only four multitarget compounds (**5**, **8**, **16** and **17**) with simultaneous activity towards H_3_R and cholinesterases were obtained in the present work. All of them are propionyl phenoxy derivatives. Unfortunately, pharmacological studies have shown that these compounds have a much greater affinity for hH_3_R (effects in the nanomolar range) than against cholinesterases (effects in the micromolar range).

In conclusion, among the series of compounds synthesized (**2**–**17**), azepane derivatives **16** and **17** proved to be the most promising ligands. Both of them can be used as lead compounds for further structural modifications. In particular, the change of length of the carbon linker (an elongation of greater than seven atoms) and/or an exchange of the ethyl substituent for a longer alkyl group (e.g., a butyl or a pentyl group) could be promising.

## 4. Materials and Methods

### 4.1. Synthesis of Compounds

Reagents and solvents were purchased from Merck and Alfa Aesar.

The course of the reaction was controlled using TLC (Merck silica 60F aluminum sheets; solvent: methylene chloride or methylene chloride: methanol 9:1). Spots were visualized under a UV lamp and/or stained with Dragendorf’s reagent. NMR spectra (^1^H and ^13^C) were obtained in DMSO-d_6_ on the following instruments: (^1^H) -Mercury 300 MHz PFG spectrometer (Varian, Palo Alto, CA, USA) and (^13^C)—FTNMR 500 MHz spectrometer (Joel Ltd., Akishima, Tokyo, Japan). Chemical shifts (δ) are given in ppm with respect to the solvent signal. Data are reported as follows: multiplicity (br s, broad singlet; d, dublet; m, multiplet; q, quartet; s, singlet; t, triplet), coupling constants (J) in Hz, and the number of protons. The mass spectrum (LC-MS) was recorded on a Waters TQ detector mass spectrometer (Water Corporation, Milford, CT, USA). Retention times (t_R_) are given in minutes. All compounds (except **6**, **11** and **17**) showed a purity of >95%. Elemental analysis was performed on an Elemental Analyser Vario El-III (Hanau, Germany). The results are in agreement with the theoretical values, within ±0.4%. Melting points (Mp) were determined on a MEL-TEMP melting point apparatus II (LD Inc., Long Beach, CA, USA), and are uncorrected.

#### 4.1.1. Synthesis of Compounds **1a**–**1d**

Bromides **1a**–**1d** were synthesized as described previously by Łażewska et al. [33], from 1-(4-hydroxyphenyl)ethan-1-one or 1-(4-hydroxyphenyl)propan-1-one, and dibromopentane or dibromohexane, respectively. After preliminary purification, crude products were used for further synthesis:

1-(4-((5-bromopentyl)oxy)phenyl)ethan-1-one CAS 61270-21-1 (**1a**)
1-(4-((6-bromohexyl)oxy)phenyl)ethan-1-one CAS138107-19-4 (**1b**)
1-(4-((5-bromopentyl)oxy)phenyl)propan-1-one (**1c**)
1-(4-((6-bromohexyl)oxy)phenyl)propan-1-one (**1d**)

#### 4.1.2. Synthesis of Compounds **2**–**17**

The compounds were obtained by reflux (12–20 h) of a proper bromide **1a**–**1d** with an amine in the mixture of ethanol:water (21:4), and in the presence of potassium carbonate and a catalytic amount of potassium iodide. The precise procedure is described in reference [33]. After purification, the oily products were transformed into salts of oxalic acid.

1-(4-((5-(Piperidin-1-yl)pentyl)oxy)phenyl)ethan-1-one hydrogen oxalate (**2**)

From 1-(4-((5-bromopentyl)oxy)phenyl)ethan-1-one (1.42 g, 5 mmol) and piperidine (0.85 g, 10 mmol). Yield 28%. Mp 117–120 °C. C_18_H_27_NO_2_ × C_2_H_2_O_4_ (MW = 379.44). Anal. calcd. for C_20_H_29_NO_6_: C, 63.30; H, 7.70; N, 3.69%. Found: C, 63.00; H, 7.57; N, 3.52%. LC-MS: purity 96.58% t_R_ = 3.72, (ESI) m/z [M+H]^+^ 290.315. ^1^H NMR (300 MHz, DMSO-d_6_) δ: 7.90 (d, *J* = 8.72 Hz, 2H), 7.01 (d, *J* = 8.72 Hz, 2H), 4.05 (t, *J* = 6.28 Hz, 2H), 2.86–3.45 (m, 5H), 2.49 (s, 3H), 1.61–1.85 (m, 8H), 1.29–1.58 (m, 4H). ^13^C NMR (126 MHz, DMSO-d_6_) δ: 196.8, 165.4, 163.0, 131.0, 130.3, 114.8, 68.0, 56.2, 52.4, 28.5, 26.9, 23.4, 23.3, 23.0, 22.1.

1-(4-((6-(Piperidin-1-yl)hexyl)oxy)phenyl)ethan-1-one hydrogen oxalate (**3**)

From 1-(4-((6-bromohexyl)oxy)phenyl)ethan-1-one (1.50 g, 5 mmol) and piperidine (0.85 g, 10 mmol). Yield 42%. Mp 116–119 °C. C_19_H_29_NO_2_ × C_2_H_2_O_4_ (MW = 393.47). Anal. calcd. for C_21_H_31_NO_6_: C, 64.10; H, 7.94; N, 3.56%. Found: C, 64.16; H, 8.18; N, 3.40%. LC-MS: purity 100% t_R_ = 4.16, (ESI) m/z [M+H]^+^ 304.329. ^1^H NMR (300 MHz, DMSO-d_6_) δ: 7.90 (d, *J* = 8.72 Hz, 2H), 7.00 (d, *J* = 8.98 Hz, 2H), 4.04 (t, *J* = 6.41 Hz, 2H), 2.83–3.38 (m, 6H), 2.49 (s, 3H), 1.58–1.78 (m, 8H), 1.21–1.54 (m, 6H). ^13^C NMR (126 MHz, DMSO-d_6_) δ: 196.8, 165.4, 163.1, 131.0, 130.3, 114.8, 68.2, 56.3, 52.4, 28.8, 26.9, 26.4, 25.5, 23.6, 23.0, 22.0.

1-(4-((5-(Piperidin-1-yl)pentyl)oxy)phenyl)propan-1-one hydrogen oxalate (**4**)

From 1-(4-((5-bromopentyl)oxy)phenyl)propan-1-one (1.50 g, 5 mmol) and 4-methylpiperidine (0.99 g, 10 mmol). Yield 41%. Mp 130–132 °C. C_19_H_29_NO_2_ × C_2_H_2_O_4_ (MW = 393.47). Anal. calcd. for C_21_H_31_NO_6_: C, 64.10; H, 7.94; N, 3.56%. Found: C, 64.34; H, 8.18; N, 3.54%. LC-MS: purity 100% t_R_ = 4.38, (ESI) m/z [M+H]^+^ 304.332. ^1^H NMR (300 MHz, DMSO-d_6_) δ: 7.91 (d, *J* = 8.72 Hz, 2H), 7.00 (d, *J* = 8.72 Hz, 2H), 4.04 (t, *J* = 6.28 Hz, 2H), 2.80–3.40 (m, 8H), 1.60–1.80 (m, 8H), 1.30–1.60 (m, 4H), 1.05 (t, *J* = 7.18 Hz, 3H). ^13^C NMR (126 MHz, DMSO-d_6_) δ 199.3, 165.4, 162.9, 130.6, 130.0, 114.9, 68.0, 56.2, 52.4, 31.3, 28.5, 23.4, 23.3, 23.0, 22.1, 8.8.

1-(4-((6-(Piperidin-1-yl)hexyl)oxy)phenyl)propan-1-one hydrogen oxalate (**5**)

From 1-(4-((6-bromohexyl)oxy)phenyl)propan-1-one (1.57 g, 5 mmol) and piperidine (0.85 g, 10 mmol). Yield 44%. Mp 120–122 °C. C_20_H_31_NO_2_ × C_2_H_2_O_4_ (MW = 407.49). Anal. calcd. for C_22_H_33_NO_6_: C, 64.84; H, 8.16; N, 3.44%. Found: C, 64.91; H, 8.13; N, 3.53%. LC-MS: purity 100% t_R_ = 4.75, (ESI) m/z [M+H]^+^ 318.353. ^1^H NMR (300 MHz, DMSO-d_6_) δ: 7.91 (d, *J* = 8.72 Hz, 2H), 7.00 (d, *J* = 8.72 Hz, 2H), 4.03 (t, *J* = 6.41 Hz, 2H), 2.81–3.38 (m, 8H), 1.22–1.86 (m, 14H), 1.04 (t, *J* = 7.18 Hz, 3H). ^13^C NMR (126 MHz, DMSO-d_6_) δ: 199.3, 165.4, 162.9, 130.6, 129.9, 114.8, 68.2, 56.3, 52.4, 31.3, 26.4, 25.5, 23.0, 22.0, 8.8.

1-(4-((5-(3-Methylpiperidin-1-yl)pentyl)oxy)phenyl)ethan-1-one hydrogen oxalate (**6**)

From 1-(4-((5-bromopentyl)oxy)phenyl)ethan-1-one (1.42 g, 5 mmol) and 3-methylpiperidine (0.99 g, 10 mmol). Yield 33%. Mp 117–120 °C. C_19_H_29_NO_2_ × C_2_H_2_O_4_ (MW = 393.47). Anal. calcd. for C_21_H_31_NO_6_: C, 64.10; H, 7.94; N, 3.56%. Found: C, 64.15; H, 7.96; N, 3.31%. LC-MS: purity 92.42% t_R_ = 4.11, (ESI) m/z [M+H]^+^ 304.340. ^1^H NMR (300 MHz, DMSO-d_6_) δ: 7.90 (d, *J* = 8.98 Hz, 2H), 7.01 (d, *J* = 8.98 Hz, 2H), 4.05 (t, *J* = 6.16 Hz, 2H), 3.23–3.43 (m, 2H), 2.88–3.03 (m, 2H), 2.69 (br. s., 1H), 2.49 (s, 3H), 2.33–2.46 (m, 1H), 1.54–1.90 (m, 8H), 1.33–1.50 (m, 2H), 1.04 (d, *J* = 11.54 Hz, 1H), 0.78–0.92 (m, 3H). ^13^C NMR (126 MHz, DMSO-d_6_) δ: 196.8, 165.4, 163.0, 131.0, 130.3, 114.8, 68.0, 57.9, 56.3, 56.2, 51.9, 30.6, 28.5, 26.9, 23.4, 23.3, 19.1.

1-(4-((6-(3-Methylpiperidin-1-yl)hexyl)oxy)phenyl)ethan-1-one hydrogen oxalate (**7**)

From 1-(4-((6-bromohexyl)oxy)phenyl)ethan-1-one (1.50 g, 5 mmol) and 3-methylpiperidine (0.99 g, 10 mmol). Yield 39%. Mp 90–93 °C. C_20_H_31_NO_2_ × C_2_H_2_O_4_ (MW = 407.47). Anal. calcd. for C_22_H_33_NO_6_: C, 64.84; H, 8.16; N, 3.44%. Found: C, 64.58; H, 8.29; N, 3.34%. LC-MS: purity 100% t_R_ = 4.53, (ESI) m/z [M+H]^+^ 318.350. ^1^H NMR (300 MHz, DMSO-d_6_) δ: 7.90 (d, *J* = 8.98 Hz, 2H), 7.00 (d, *J* = 8.98 Hz, 2H), 4.04 (t, *J* = 6.28 Hz, 2H), 3.31 (t, *J* = 14.11 Hz, 2H), 2.83–3.03 (m, 2H), 2.68 (br. s., 1H), 2.49 (s, 3H), 2.31–2.45 (m, 1H), 1.54–1.96 (m, 8H), 1.22–1.51 (m, 4H), 0.93–1.12 (m, 1H), 0.86 (d, *J* = 6.67 Hz, 3H). ^13^C NMR (126 MHz, DMSO-d_6_) δ: 196.8, 165.3, 163.1, 131.0, 130.3, 114.8, 68.2, 57.9, 56.3, 51.9, 30.6, 28.8, 26.9, 26.4, 25.5, 23.6, 22.5, 19.1.

1-(4-((5-(3-Methylpiperidin-1-yl)pentyl)oxy)phenyl)propan-1-one hydrogen oxalate (**8**)

From 1-(4-((5-bromopentyl)oxy)phenyl)propan-1-one (1.5 g, 5 mmol) and 3-methylpiperidine (0.99 g, 10 mmol). Yield 42%. Mp 132–134 °C. C_20_H_31_NO_2_ × C_2_H_2_O_4_ (MW = 407.49). Anal. calcd. for C_22_H_33_NO_6_: C, 64.84; H, 8.16; N, 3.44%. Found: C, 64.80; H, 8.43; N, 3.54%. LC-MS: purity 100% t_R_ = 4.74, (ESI) m/z [M+H]^+^ 318.349. ^1^H NMR (300 MHz, DMSO-d_6_) δ: 7.91 (d, *J* = 8.72 Hz, 2H), 7.00 (d, *J* = 8.72 Hz, 2H), 4.04 (t, *J* = 6.28 Hz, 2H), 3.21–3.42 (m, 2H), 2.86–3.07 (m, 4H), 2.60–2.78 (m, 1H), 2.34–2.45 (m, 1H), 1.53–1.97 (m, 8H), 1.31–1.52 (m, 2H), 1.05 (t, *J* = 7.18 Hz, 4H), 0.86 (d, *J* = 6.41 Hz, 3H). ^13^C NMR (126 MHz, DMSO-d_6_) δ: 199.33, 165.34, 162.87, 130.62, 129.97, 114.78, 68.0, 57.88, 56.27, 51.93, 31.30, 30.62, 28.95, 28.52, 23.40, 23.26, 22.69, 19.13, 8.84.

1-(4-((6-(3-Methylpiperidin-1-yl)hexyl)oxy)phenyl)propan-1-one hydrogen oxalate (**9**)

From 1-(4-((6-bromohexyl)oxy)phenyl)propan-1-one (1.57 g, 5 mmol) and 3-methylpiperidine (0.99 g, 10 mmol). Yield 36%. Mp 77–80 °C. C_21_H_33_NO_2_ × C_2_H_2_O_4_ (MW = 421.52). Anal. calcd. for C_23_H_35_NO_6_: C, 65.53; H, 8.37; N, 3.32%. Found: C, 65.68; H, 8.35; N, 3.43%. LC-MS: purity 100% t_R_ = 5.07, (ESI) m/z [M+H]^+^ 332.382. ^1^H NMR (300 MHz, DMSO-d_6_) δ: 7.91 (d, *J* = 8.98 Hz, 2H), 7.00 (d, *J* = 8.98 Hz, 2H), 4.03 (t, *J* = 6.28 Hz, 2H), 3.21–3.41 (m, 2H), 2.82–3.03 (m, 4H), 2.68 (t, *J* = 10.39 Hz, 1H), 2.34–2.45 (m, 1H), 1.54–1.92 (m, 8H), 1.19–1.51 (m, 4H), 1.04 (t, *J* = 7.18 Hz, 4H), 0.86 (d, *J* = 6.41 Hz, 3H). ^13^C NMR (126 MHz, DMSO-d_6_) δ: 199.3, 165.3, 162.9, 130.6, 129.9, 114.8, 68.2, 57.9, 56.4, 51.9, 31.3, 30.6, 29.0, 28.8, 26.4, 25.5, 23.6, 22.7, 19.1, 8.8.

1-(4-((5-(4-Methylpiperidin-1-yl)pentyl)oxy)phenyl)ethan-1-one hydrogen oxalate (**10**)

From 1-(4-((5-bromopentyl)oxy)phenyl)ethan-1-one (1.42 g, 5 mmol) and 4-methylpiperidine (0.99 g, 10 mmol). Yield 32%. Mp 122–125 °C. C_19_H_29_NO_2_ x C_2_H_2_O_4_ (MW = 393.47). Anal. calcd. for C_21_H_31_NO_6_: C, 64.10; H, 7.94; N, 3.56%. Found: C, 63.75; H, 7.85; N, 3.54%. LC-MS: purity 100% t_R_ = 4.12 (ESI) m/z [M+H]^+^ 304.332. ^1^H NMR (300 MHz, DMSO-d_6_) δ: 7.90 (d, *J* = 8.72 Hz, 2H), 7.01 (d, *J* = 8.72 Hz, 2H), 4.05 (t, *J* = 6.28 Hz, 2H), 3.28–3.46 (m, 2H), 2.90–3.05 (m, 2H), 2.81 (t, *J* = 11.41 Hz, 2H), 2.49 (s, 3H), 1.51–1.83 (m, 7H), 1.21–1.48 (m, 4H), 0.90 (d, *J* = 6.41 Hz, 3H). ^13^C NMR (126 MHz, DMSO-d6) δ: 196.80, 165.34, 163.01, 131.02, 130.32, 114.77, 68.03, 55.94, 51.87, 30.99, 28.50, 26.93, 23.53, 23.25, 21.38.

1-(4-((6-(4-Methylpiperidin-1-yl)hexyl)oxy)phenyl)ethan-1-one hydrogen oxalate (**11**)

From 1-(4-((6-bromohexyl)oxy)phenyl)ethan-1-one (1.50 g, 5 mmol) and 4-methylpiperidine (0.99 g, 10 mmol). Yield 48%. Mp 111–114 °C. C_20_H_31_NO_2_ × C_2_H_2_O_4_ (MW = 407.47). Anal. calcd. for C_22_H_33_NO_6_: C, 64.84; H, 8.16; N, 3.44%. Found: C, 65.79; H, 8.70; N, 3.22%. LC-MS: purity 94.57% t_R_ = 4.45, (ESI) m/z [M+H]^+^ 318.359. ^1^H NMR (300 MHz, DMSO-d_6_) δ: 7.90 (d, *J* = 8.72 Hz, 2H), 7.00 (d, *J* = 8.72 Hz, 2H), 4.04 (t, *J* = 6.28 Hz, 2H), 3.24–3.46 (m, 2H), 2.68–3.03 (m, 4H), 2.49 (s, 3H), 1.20–1.85 (m, 13H), 0.90 (d, *J* = 6.41 Hz, 3H). ^13^C NMR (126 MHz, DMSO-d_6_) δ: 196.8, 165.3, 163.1, 131.0, 130.3, 114.8, 68.2, 28.8, 26.9, 26.4, 25.5, 23.7.

1-(4-((5-(4-Methylpiperidin-1-yl)pentyl)oxy)phenyl)propan-1-one hydrogen oxalate (**12**)

From 1-(4-((5-bromopentyl)oxy)phenyl)propan-1-one (1.50 g, 5 mmol) and 4-methylpiperidine (0.99 g, 10 mmol). Yield 39%. Mp 142–145 °C. C_20_H_31_NO_2_ × C_2_H_2_O_4_ (MW = 407.49). Anal. calcd. for C_22_H_33_NO_6_: C, 64.84; H, 8.16; N, 3.44%. Found: C, 64.97; H, 8.48; N, 3.49%. LC-MS: purity 98.17% t_R_ = 4.54, (ESI) m/z [M+H]^+^ 318.366. ^1^H NMR (300 MHz, DMSO-d_6_) δ: 7.90 (d, *J* = 8.72 Hz, 2H), 7.00 (d, *J* = 8.72 Hz, 2H), 4.04 (t, *J* = 6.28 Hz, 2H), 3.35 (d, *J* = 11.54 Hz, 2H), 2.95 (q, *J* = 7.18 Hz, 4H), 2.81 (t, *J* = 11.54 Hz, 2H), 1.51–1.88 (m, 7H), 1.27–1.49 (m, 4H), 1.04 (t, *J* = 7.18 Hz, 3H), 0.83–0.94 (m, 3H). ^13^C NMR (126 MHz, DMSO-d_6_) δ: 199.3, 165.4, 162.9, 130.6, 130.0, 114.8, 68.0, 55.9, 51.9, 31.3, 31.0, 28.5, 23.5, 23.3, 21.4, 8.8.

1-(4-((6-(4-Methylpiperidin-1-yl)hexyl)oxy)phenyl)propan-1-one hydrogen oxalate (**13**)

From 1-(4-((6-bromohexyl)oxy)phenyl)propan-1-one (1.57 g, 5 mmol) and 4-methylpiperidine (0.99 g, 10 mmol). Yield 38%. Mp 115–117 °C. C_21_H_33_NO_2_ × C_2_H_2_O_4_ (MW = 421.52). Anal. calcd. for C_23_H_35_NO_6_: C, 65.53; H, 8.37; N, 3.32%. Found: C, 65.59; H, 8.29; N, 3.41%. LC-MS: purity 100% t_R_ = 5.12, (ESI) m/z [M+H]^+^ 332.372. ^1^H NMR (300 MHz, DMSO-d_6_) δ: 7.91 (d, *J* = 8.72 Hz, 2H), 7.00 (d, *J* = 8.72 Hz, 2H), 4.03 (t, *J* = 6.28 Hz, 2H), 3.23–3.47 (m, 2H), 2.87–3.04 (m, 4H), 2.80 (t, *J* = 11.54 Hz, 2H), 1.48–1.86 (m, 7H), 1.16–1.45 (m, 6H), 1.04 (t, *J* = 7.18 Hz, 3H), 0.71–0.93 (m, 3H). ^13^C NMR (126 MHz, DMSO-d_6_) δ: 199.3, 165.3, 162.9, 130.6, 129.9, 114.8, 68.2, 56.0, 51.9, 31.3, 31.0, 28.8, 28.5, 26.4, 25.5, 23.7, 21.4, 8.8.

1-(4-((5-(Azepan-1-yl)pentyl)oxy)phenyl)ethan-1-one hydrogen oxalate (**14**)

From 1-(4-((5-bromopentyl)oxy)phenyl)ethan-1-one (1.42 g, 5 mmol) and azepane (0.99 g, 10 mmol). Yield 24%. Mp 102–105 °C. C_19_H_29_NO_2_ × C_2_H_2_O_4_ (MW = 393.47). Anal. calcd. for C_21_H_31_NO_6_: C, 64.10; H, 7.94; N, 3.56%. Found: C, 63.77; H, 7.94; N, 3.57%. LC-MS: purity 97.81% t_R_ = 4.04, (ESI) m/z [M+H]^+^ 304.336. ^1^H NMR (300 MHz, DMSO-d_6_) δ: 7.90 (d, *J* = 8.72 Hz, 2H), 7.01 (d, *J* = 8.72 Hz, 2H), 4.05 (t, *J* = 6.28 Hz, 2H), 3.18 (br. s., 4H), 2.94–3.08 (m, 2H), 2.49 (s, 3H), 1.51–1.86 (m, 12H), 1.32–1.49 (m, 2H). ^13^C NMR (126 MHz, DMSO-d_6_) δ 196.8, 165.4, 163.0, 131.0, 130.3, 114.8, 68.1, 56.6, 54.0, 28.5, 26.9, 26.6, 23.8, 23.5, 23.3.

1-(4-((6-(Azepan-1-yl)hexyl)oxy)phenyl)ethan-1-one hydrogen oxalate (**15**)

From 1-(4-((6-bromohexyl)oxy)phenyl)ethan-1-one (1.50 g, 5 mmol) and azepane (0.99 g, 10 mmol). Yield 38%. Mp 91–94 °C. C_20_H_31_NO_2_ × C_2_H_2_O_4_ (MW = 407.47). Anal. calcd. for C_22_H_33_NO_6_: C, 64.84; H, 8.16; N, 3.44%. Found: C, 64.75; H, 8.36; N, 3.28%. LC-MS: purity 100% t_R_ = 4.46, (ESI) m/z [M+H]^+^ 318.351. ^1^H NMR (300 MHz, DMSO-d_6_) δ:7.90 (d, *J* = 8.72 Hz, 2H), 7.00 (d, *J* = 8.72 Hz, 2H), 4.04 (t, *J* = 6.28 Hz, 2H), 3.16 (d, *J* = 4.36 Hz, 4H), 2.93–3.06 (m, 2H), 2.49 (s, 3H), 1.69–1.86 (m, 6H), 1.51–1.68 (m, 6H), 1.23–1.49 (m, 4H). ^13^C NMR (126 MHz, DMSO-d_6_) δ: 196.8, 165.4, 163.1, 131.0, 130.3, 114.8, 68.2, 56.7, 53.9, 28.8, 26.9, 26.6, 26.4, 25.5, 24.0, 23.5.

1-(4-((5-(Azepan-1-yl)pentyl)oxy)phenyl)propan-1-one hydrogen oxalate (**16**)

From 1-(4-((5-bromopentyl)oxy)phenyl)propan-1-one (1.50 g, 5 mmol) and azepane (0.99 g, 10 mmol). Yield 32%. Mp 132–134 °C. C_20_H_31_NO_2_ × C_2_H_2_O_4_ (MW = 407.49). Anal. calcd. for C_22_H_33_NO_6_: C, 64.84; H, 8.16; N, 3.44%. Found: C, 64.66; H, 8.33; N, 3.44%. LC-MS: purity 98.59% t_R_ = 4.68, (ESI) m/z [M+H]^+^ 318.350. ^1^H NMR (300 MHz, DMSO-d_6_) δ: 7.91 (d, *J* = 8.72 Hz, 2H), 7.00 (d, *J* = 8.72 Hz, 2H), 4.05 (t, *J* = 6.41 Hz, 2H), 3.18 (br s., 4H), 2.87–3.08 (m, 4H), 1.63–1.85 (m, 8H), 1.58 (br. s., 4H), 1.32–1.49 (m, 2H), 1.05 (t, *J* = 7.18 Hz, 3H). ^13^C NMR (126 MHz, DMSO-d_6_) δ: 199.33, 165.41, 162.87, 130.62, 129.98, 114.78, 68.03, 56.60, 53.94, 31.30, 28.52, 26.64, 23.82, 23.46, 23.26, 8.84.

1-(4-((6-(Azepan-1-yl)hexyl)oxy)phenyl)propan-1-one hydrogen oxalate (**17**)

From 1-(4-((6-bromohexyl)oxy)phenyl)propan-1-one (1.57 g, 5 mmol) and azepane (0.99 g, 10 mmol). Yield 41%. Mp 98–100 °C. C_21_H_33_NO_2_ × C_2_H_2_O_4_ (MW = 421.52). Anal. calcd. for C_23_H_35_NO_6_: C, 65.53; H, 8.37; N, 3.32%. Found: C, 65.63; H, 8.54; N, 3.29%. LC-MS: purity 94.76% t_R_ = 5.11, (ESI) m/z [M+H]^+^ 332.374. ^1^H NMR (300 MHz, DMSO-d_6_) δ: 7.91 (d, *J* = 8.72 Hz, 2H), 7.00 (d, *J* = 8.72 Hz, 2H), 4.03 (t, *J* = 6.41 Hz, 2H), 3.17 (br s, 4H), 2.86-3.05 (m, 4H), 1.49-1.87 (m, 12H), 1.20-1.46 (m, 4H), 1.04 (t, *J* = 7.18 Hz, 3H). ^13^C NMR (126 MHz, DMSO-d_6_) δ: 199.32, 165.40, 162.91, 130.62, 129.94, 114.76, 68.18, 56.69, 53.92, 31.30, 28.79, 26.63, 26.37, 25.52, 24.02, 23.45, 8.84.

### 4.2. Biological Studies

Compounds **2**–**17** were tested as oxalic acid salts.

#### 4.2.1. Histamine H_3_ Receptor Affinity

H_3_R receptor affinity assays were performed in the radioligand competition assay. Human H_3_R was stably expressed in HEK293 cells. [^3^H]-*N*^α^-Methylhistamine (K_D_ = 3.08 nM) was used as the radioligand. The compounds used in the experiments were dissolved in DMSO. The precise experiment was performed as previously described [30]. Complete curves were obtained for at least seven approximate concentrations, in at least three independent experiments, performed at least in duplicate. *K_i_* values were calculated using the Cheng–Prusoff equation. Results are presented as mean values with confidence intervals (CI) (95%).

#### 4.2.2. Cholinesterase Inhibitory Activity

AChE from *electrophorus electricus* and BuChE from *equine serum* were purchased from Sigma-Aldrich (Steinheim, Germany). The whole procedure was performed as previously described, using a modified Ellman method in 96-well microplates [19]. Absorbance was measured using an EnSpire multimode microplate reader (PerkinElmer, Waltham, MA, USA) at 412 nm. Initial screening was performed for compounds at a concentration of 10 micromoles. The percentage of inhibition was calculated by comparison with a blank sample (100% enzyme activity). For compounds showing >70% inhibition of enzyme activity, IC_50_ values were determined, measuring absorbance at six different inhibitor concentrations. Each concentration was measured in triplicate. IC_50_ values were calculated using GrapPad Prism 9 software (GraphPad Software, San Diego, CA, USA). Data are expressed as mean ± SEM.

#### 4.2.3. Kinetic Studies of Cholinesterases

Studies were performed with compounds **16** and **17** using a method as described previously [19]. Vmax and Km values of the Michaelis–Menten kinetics were calculated by nonlinear regression from substrate–velocity curves. Lineweaver–Burk and Cornish–Bowden plots were calculated using linear regression in GraphPad Prism 9 software (GraphPad Software, San Diego, CA, USA). Each experiment was performed in triplicate.

#### 4.2.4. Monoamine Oxidase B Inhibitory Activity

The compounds were evaluated by the spectrophotometric method described earlier [2032]. The enzyme was purchased from Sigma-Aldrich (Steinheim, Germany). Screening was carried out at a concentration of 1 micromolar. Inhibitor activity was measured in the presence of p-tyramine. The percentage inhibition of reference compounds, pargiline and rasagiline, tested at 1 micromolar, were 95 and 100%, respectively.

Data are expressed as mean values of two independent experiments.

#### 4.2.5. Toxicity Evaluation

The hepatoma cell line HepG2 (ATCC^®^ HB-8065TM) and SH-SY5Y CRL-2266 neuroblastoma cell line were used to evaluate the toxicity of tested compounds. Tests were conducted as described previously [33]. The CellTiter 96^®^ Aqueous Non-Radioactive Cell Proliferation Assay was purchased from Promega (Madison, WI, USA). Compounds were tested at 7 concentrations (0.78, 1.56, 3.125, 6.25, 12.5, 25 and 50 µM). Cell viability was determined after incubation with compounds for 72 h. Each experiment was performed twice, in triplicate.

## Figures and Tables

**Table 1:** Histamine H_3_ receptor affinities, cholinesterases, and MAO B inhibitory activity of the tested compounds.

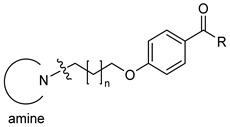							
Comp	Amine	n	R	hH_3_R ^a^*K_i_* (nM) Mean [CI 95%]	*ee*AChE ^b^ IC_50_ (µM) Mean ± SEM (% inh) ^e^	*eq*BuChE ^c^ IC_50_ (µM) Mean ± SEM (% inh) ^e^	hMAO B ^d^ (% inh) ^f^
**2**	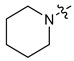	3	CH_3_	34 [24;47]	(45%)	(25%)	(2%)
**3**		4	CH_3_	75 [34;169]	(67%)	(23%)	(3%)
**4**		3	C_2_H_5_	24 [11;54]	(49%)	(32%)	(16%)
**5**		4	C_2_H_5_	41 [27;64]	4.79 ± 0.19	1.35 ± 0.04	(22%)
**6**	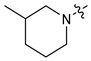	3	CH_3_	26 [13;52]	3.06 ± 0.10	(14%)	(10%)
**7**		4	CH_3_	72 [41;127]	1.75 ± 0.05	(18%)	(0%)
**8**		3	C_2_H_5_	12 [6;22]	2.10 ± 0.06	1.75 ± 0.05	(19%)
**9**		4	C_2_H_5_	38 [20;72]	(68%)	(66%)	(12%)
**10**	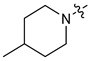	3	CH_3_	120 [82;177]	(60%)	(43%)	(0%)
**11**		4	CH_3_	106 [76;149]	2.65 ± 0.11	(49%)	(0%)
**12**		3	C_2_H_5_	57 [36;91]	(58%)	1.29 ± 0.08	(0%)
**13**		4	C_2_H_5_	58 [35;96]	2.56 ± 0.08	(65%)	(1%)
**14**	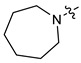	3	CH_3_	52 [38;71]	(63%)	(63%)	(37%)
**15**		4	CH_3_	79 [41;150]	(67%)	(68%)	(6%)
**16**		3	C_2_H_5_	30 [17;53]	3.60 ± 0.12	0.55 ± 0.02	(6%)
**17**		4	C_2_H_5_	42 [20;85]	1.06 ± 0.03	2.86 ± 0.03	(5%)
**pitolisant**				12 ± 3 ^g^	(3%) ^g^	8.42 ± 0.18 ^g^	(2%) ^g^
**donepezil**				nt ^h^	0.04 ± 0.01 ^g^	1.83 ± 0.02	nt ^h^
**safinamide**				nt ^h^	nt ^h^	nt ^h^	(98%) 7.60 ± 1.0 ^i^

^a^ [^3^H]-*N*^α^-Methylhistamine binding assay performed in HEK293 cells expressing the human H_3_ receptor; mean value with a confidence interval (CI95%) from at least three independent repetitions ^b^ AChE of *electric eel*; IC_50_, mean value of three independent experiments; ^c^ BuChE of *equine serum*; IC_50_, mean value of three independent experiments. ^d^ Fluorometric Amplex^TM^ red MAO assay. ^e^ Percentage inhibition of *ee*AChE or *eq*BuChE at 10 μM. ^f^ Percentage inhibition of hMAO B at 1 μM; mean values of two independent experiments. ^g^ Data from Ref. [19]. ^h^ Not tested. ^i^ IC_50_ in nM; data from ref. [20].

**Table 2:** Summary of toxicity evaluation of selected compounds in MTS assay.

Compound	HepG2 ^a^ IC_50_ [µM]	Cell Viability (%) ^b^	SH-SY5Y ^a^ IC_50_ [µM]	Cell Viability (%) ^b^
**5**	158	69.55 ± 2.14	-	No toxic effect
**6**	120	74.32 ± 5.58	149	86.23 ± 2.20
**7**	148	72.38 ± 4.54	-	No toxic effect
**8**	142	70.89 ± 2.92	349	90.73 ± 1.50
**11**	223	77.52 ± 0.94	-	No toxic effect
**12**	149	77.20 ± 4.14	421	90.70 ± 12.88
**13**	105	64.66 ± 4.40	-	No toxic effect
**16**	92	67.69 ± 1.14	357	90.28 ± 5.32
**17**	60	58.02 ± 3.54	111	70.35 ± 4.46

^a^ The IC_50_ value from the MTS assay in HepG2 and SH-SY5Y cells, at 48 h and 24 h of exposure, respectively. IC_50_ values were calculated with a non-linear regression fit to a sigmoidal dose–response curve (log of compound concentration vs. normalized response) using GraphPad Prism (v. 4.0.3). ^b^ Cell viability at the concentration of 50 μM, expressed as the percentage of control (vehicle-treated cells), set as 100%.

## Data Availability

Not applicable.

## Supplementary Materials

The following supporting information can be downloaded at: https://www.mdpi.com/article/10.3390/molecules28052349/s1, Spectral information (^1^H-NMR and ^13^C-NMR) of synthesized compounds and Figures S1 and S2 (toxicity evaluation in HepG2 and SH-SY5Y cells).

## Sample Availability

Samples of the compounds could be available from the authors.
