# Supplementary material for: Multitargeting Histamine H3 Receptor Ligands among Acetyl- and Propionyl-Phenoxyalkyl Derivatives
Source: Molecules. 2023 Mar 3;28(5):2349. doi: 10.3390/molecules28052349 (PMC10005104; doi:10.3390/molecules28052349)
Supplement: Supplementary file 1 [file molecules-28-02349-s001.zip › Lazewska - suppl 1H & 13C NMR.pdf]

Multitargeting histamine H_3 receptor ligands among acetyl- and propionyl-phenoxyalkyl derivatives

Dorota Łażewska[1*], Maria Kaleta[1], Paula Zaręba[2], Justyna Godyń[2], Mariam Dubiel[3], Ewelina Honkisz-Orzechowska[1], Agata Doroz-Płonka[1], Anna Więckowska[2], Holger Stark[3] and Katarzyna Kieć-Kononowicz[1]

[1] Department of Technology and Biotechnology of Drugs, Faculty of Pharmacy, Jagiellonian University Medical College in Kraków, Medyczna 9, 30-688 Krakow, Poland;
[2] Department of Physicochemical Drug Analysis, Jagiellonian University Medical College in Kraków, Medyczna 9, 30-688 Krakow, Poland
[3] Institute of Pharmaceutical and Medicinal Chemistry, Heinrich Heine University Düsseldorf, Universitaetsstr. 1, 40225 Duesseldorf, Germany

*** Correspondence:** dorota.lazewska@uj.edu.pl;

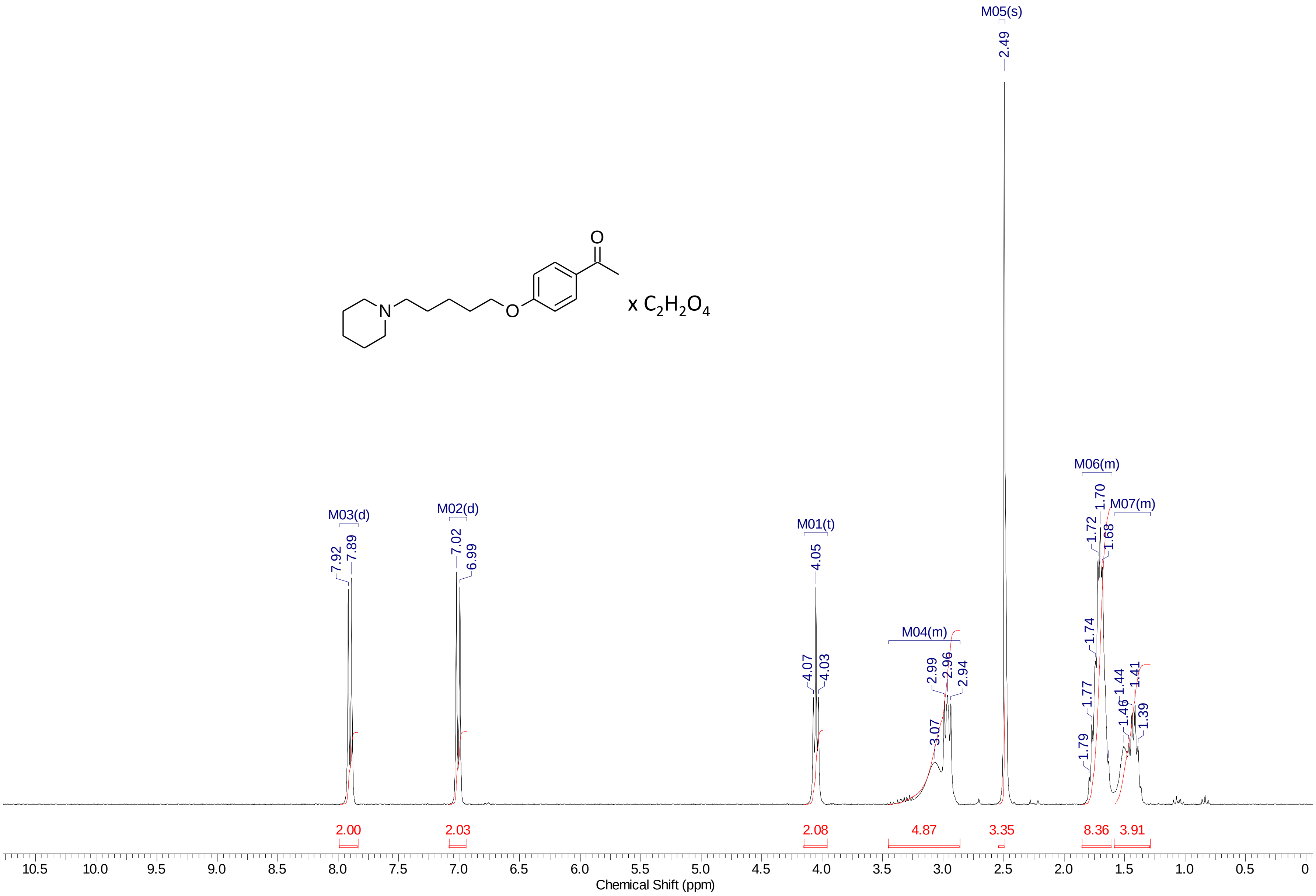

Figure S1. ^{1}H NMR spectrum of 1-(4-((-(piperidin-1-yl)pentyl)oxy)phenyl)ethan-1-one hydrogen oxalate (**2**)

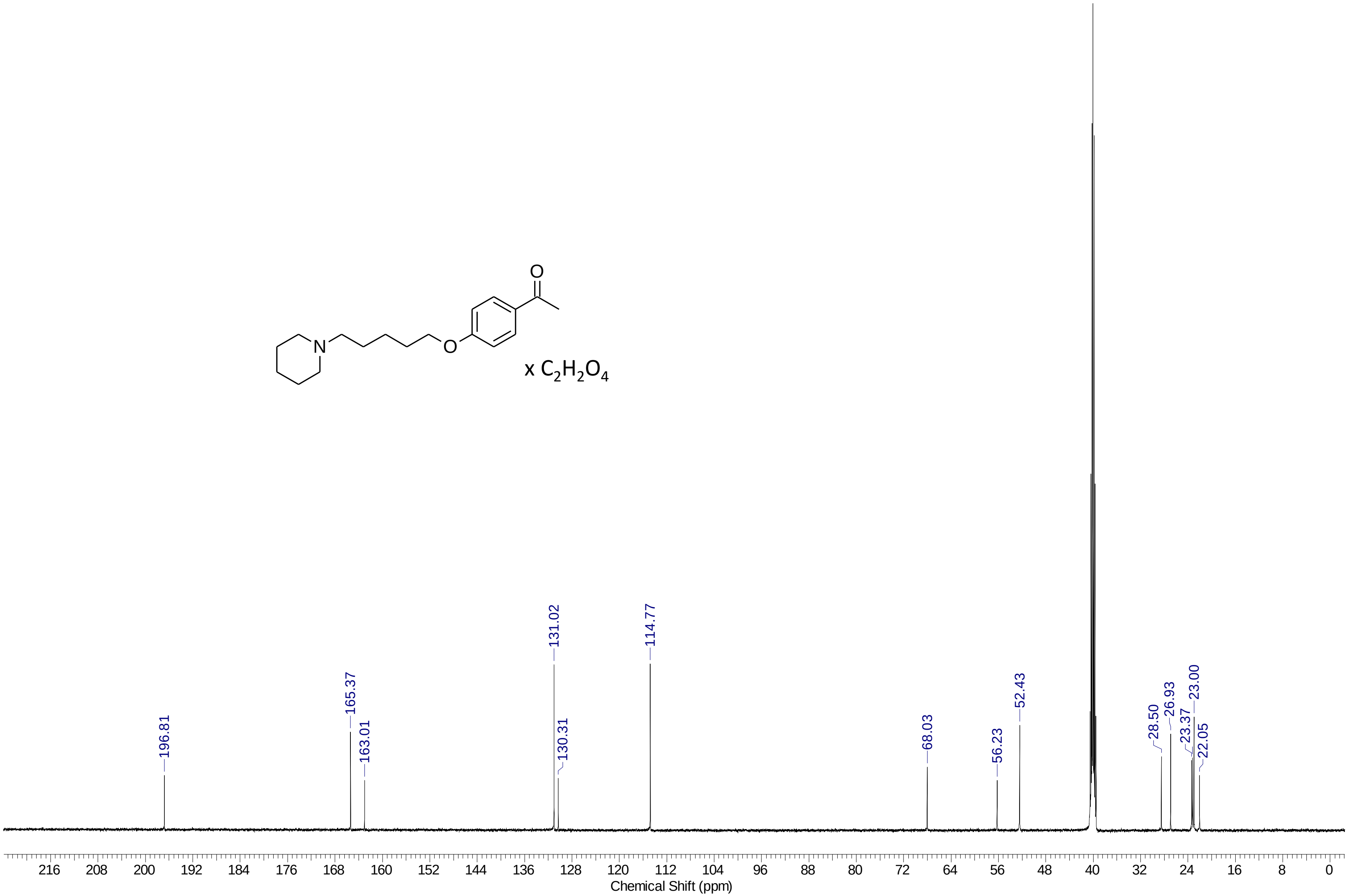

Figure S2. ^{13}C NMR spectrum of 1-(4-((5-(piperidin-1-yl)pentyl)oxy)phenyl)ethan-1-one hydrogen oxalate (**2**)

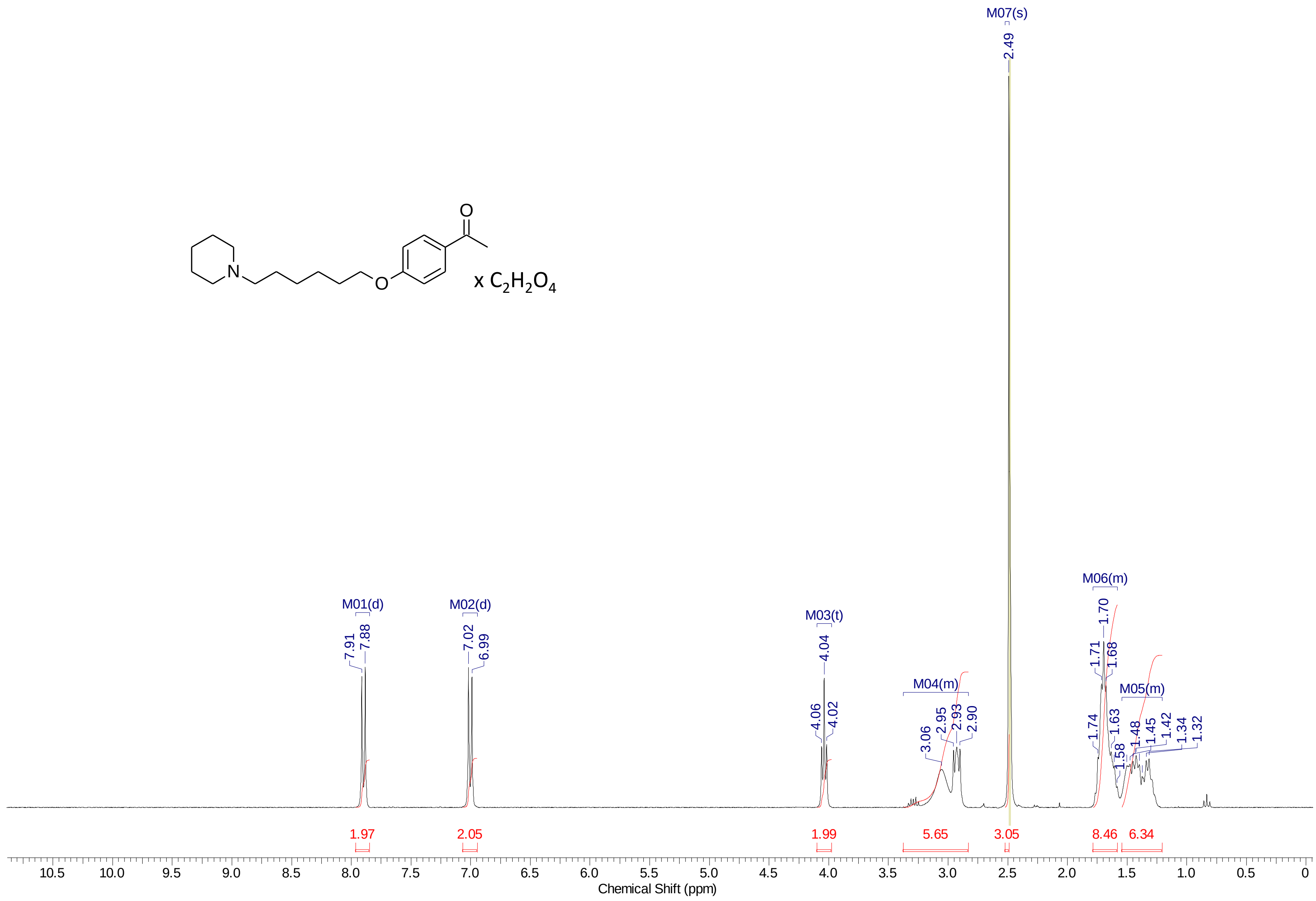

Figure S3. ^{1}H NMR spectrum of 1-(4-((6-(piperidin-1-yl)hexyl)oxy)phenyl)ethan-1-one hydrogen oxalate (**3**)

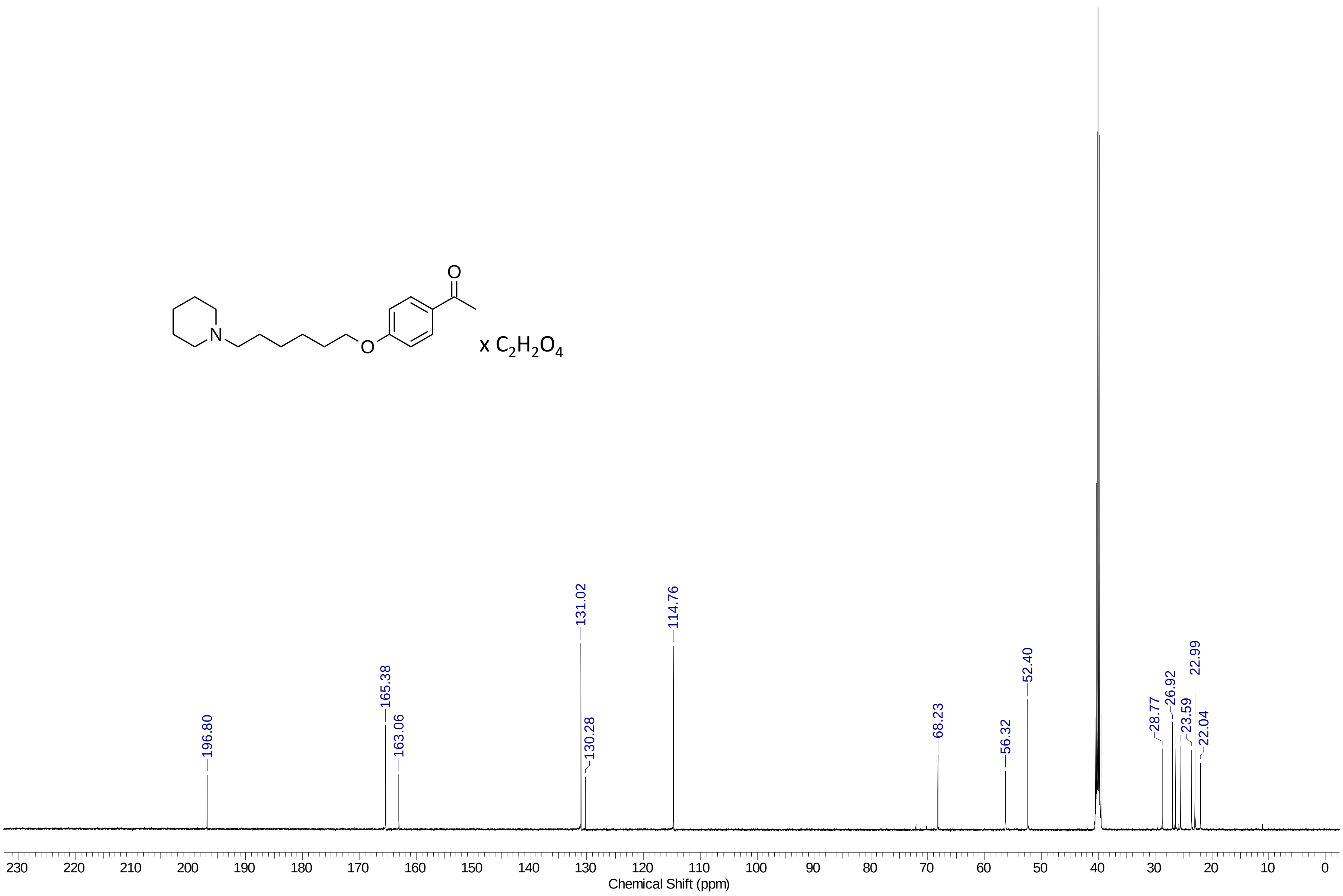

Figure S4. ^{13}C NMR spectrum of 1-(4-((6-(piperidin-1-yl)hexyl)oxy)phenyl)ethan-1-one hydrogen oxalate (**3**)

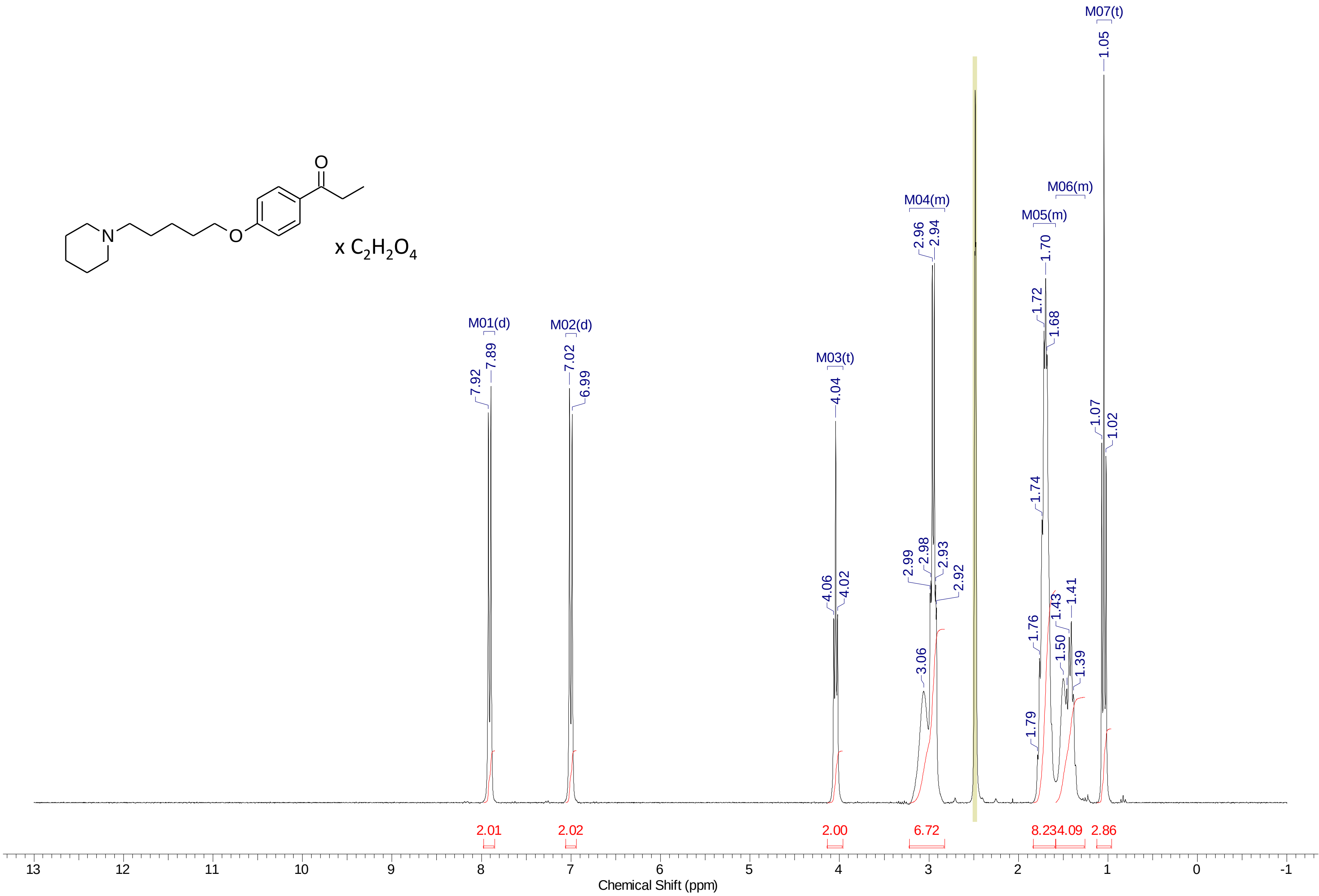

Figure S5. ^{1}H NMR spectrum of 1-(4-((5-(piperidin-1-yl)pentyl)oxy)phenyl)ethan-1-one hydrogen oxalate (**4**)

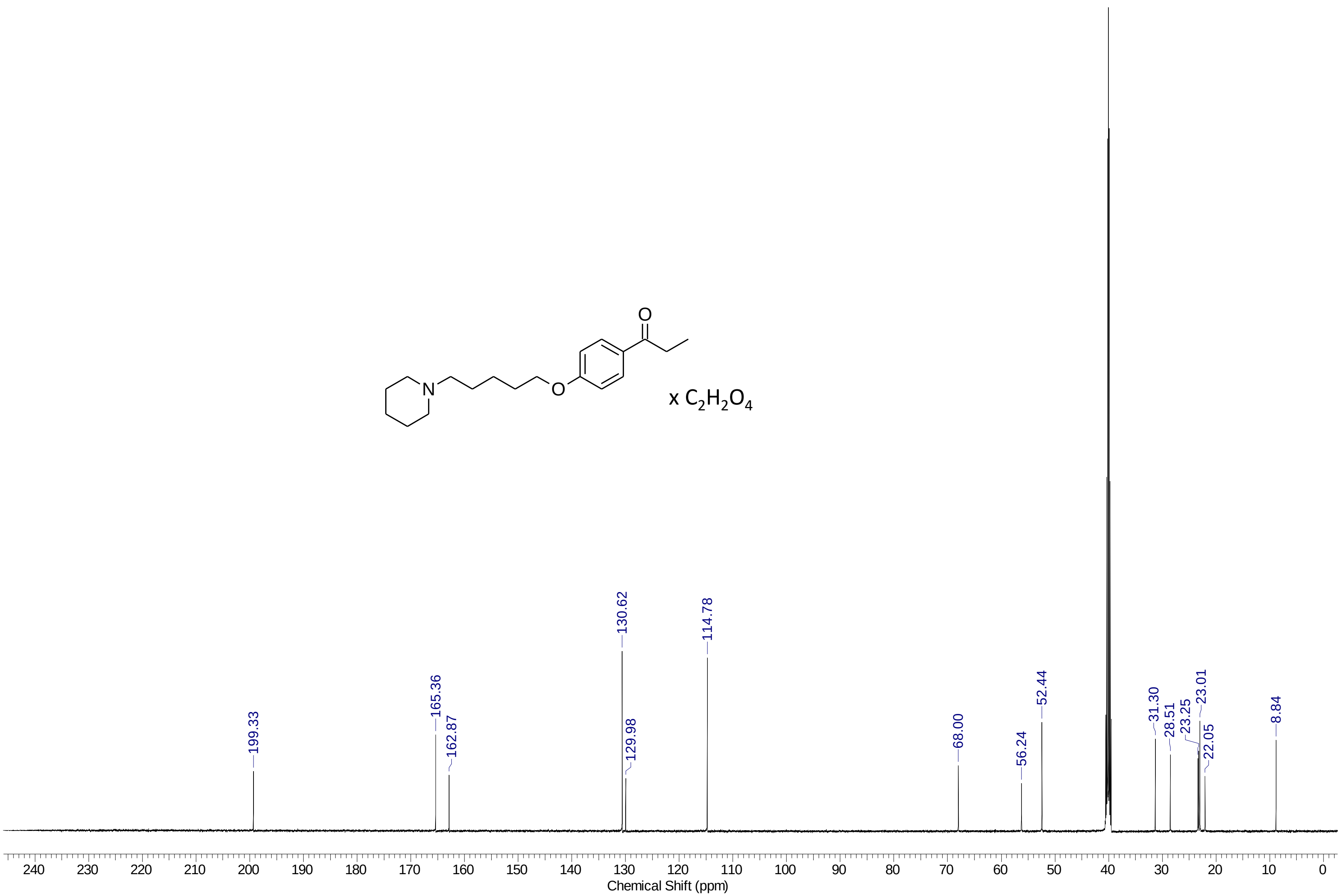

Figure S6. ^{13}C NMR spectrum of 1-(4-((5-(piperidin-1-yl)pentyl)oxy)phenyl)ethan-1-one hydrogen oxalate (**4**)

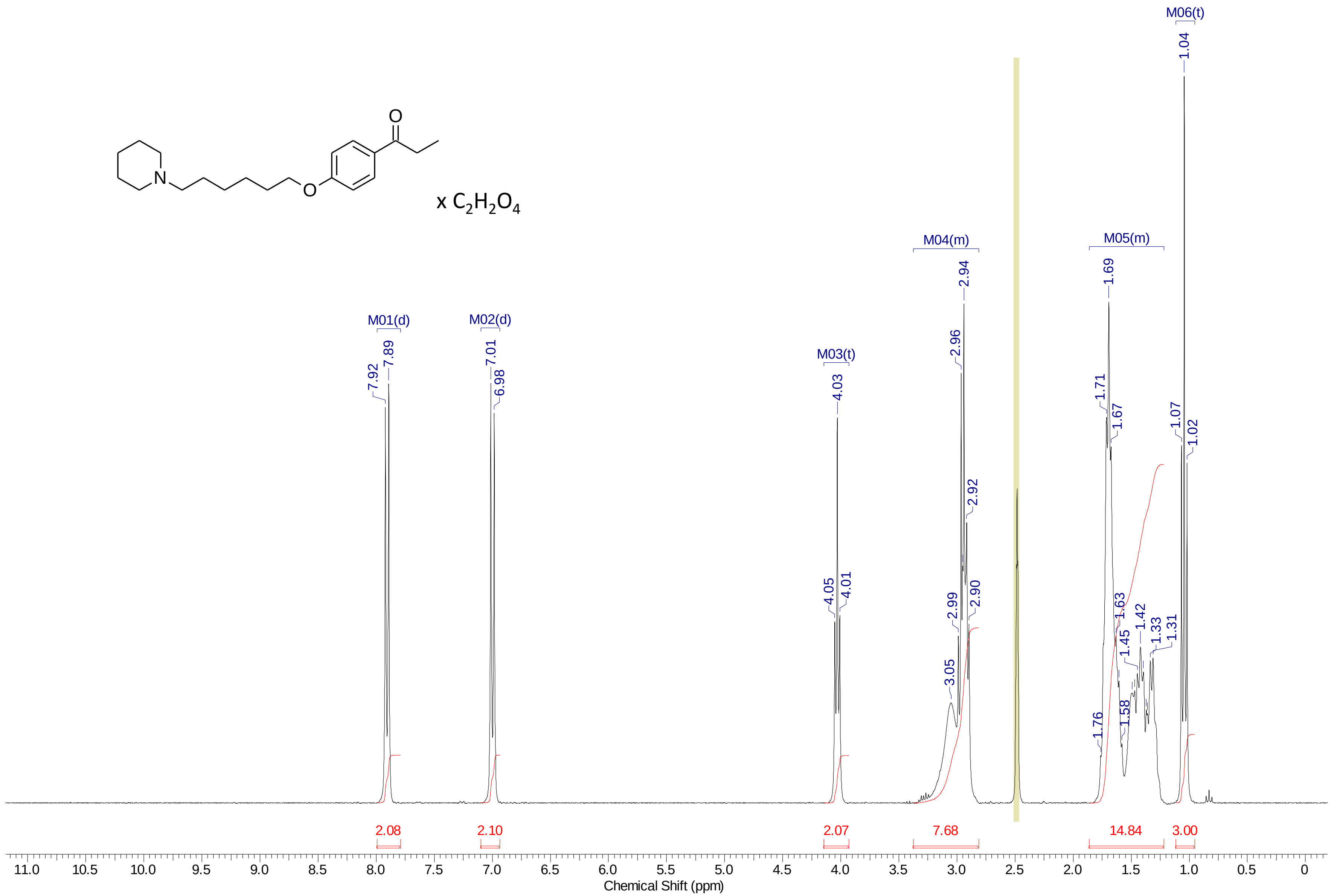

Figure S7. ^{1}H NMR spectrum of 1-(4-((6-(piperidin-1-yl)hexyl)oxy)phenyl)propan-1-one hydrogen oxalate (**5**)

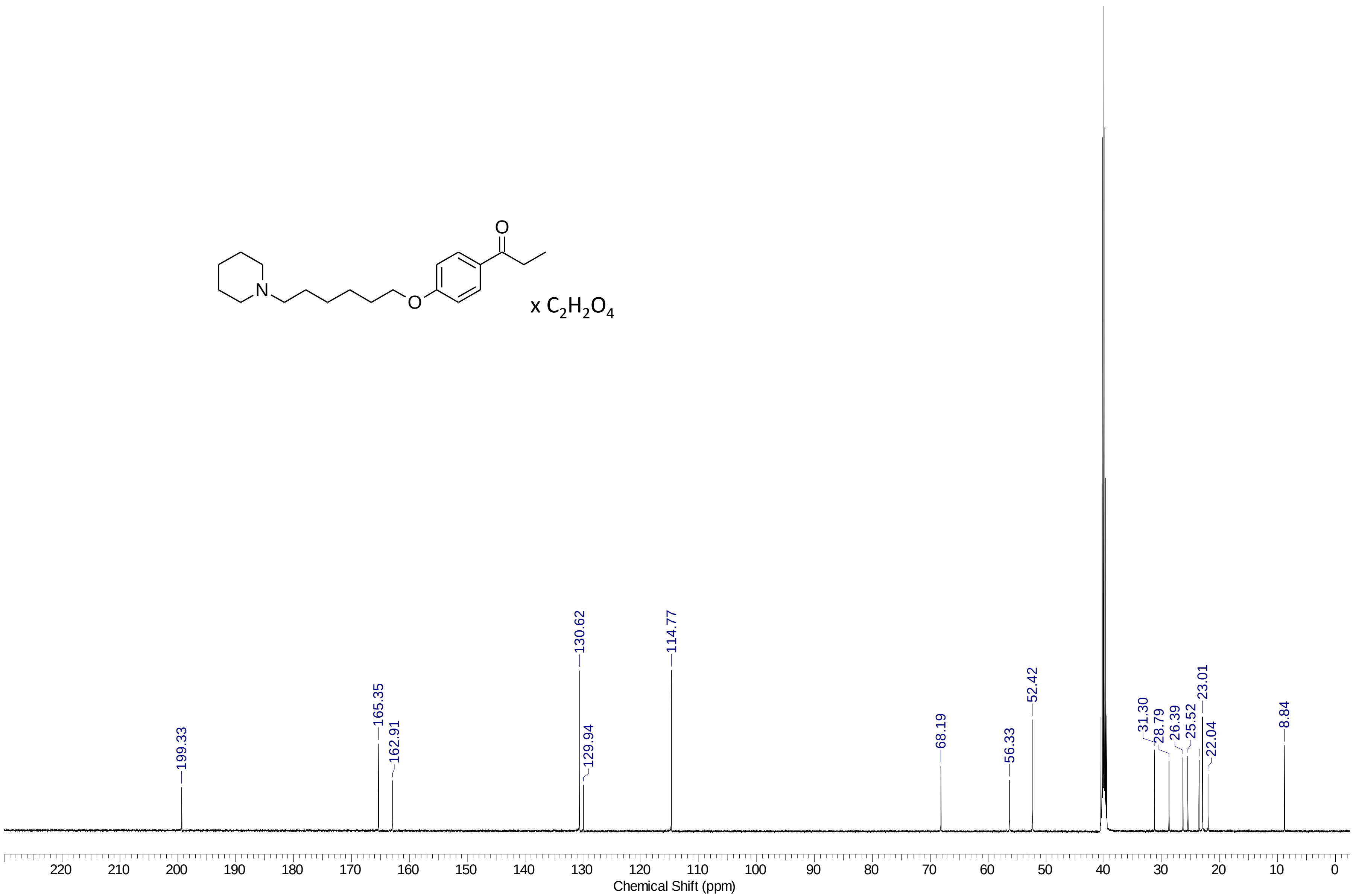

Figure S8. ^{13}C NMR spectrum of 1-(4-((6-(piperidin-1-yl)hexyl)oxy)phenyl)propan-1-one hydrogen oxalate (**5**)

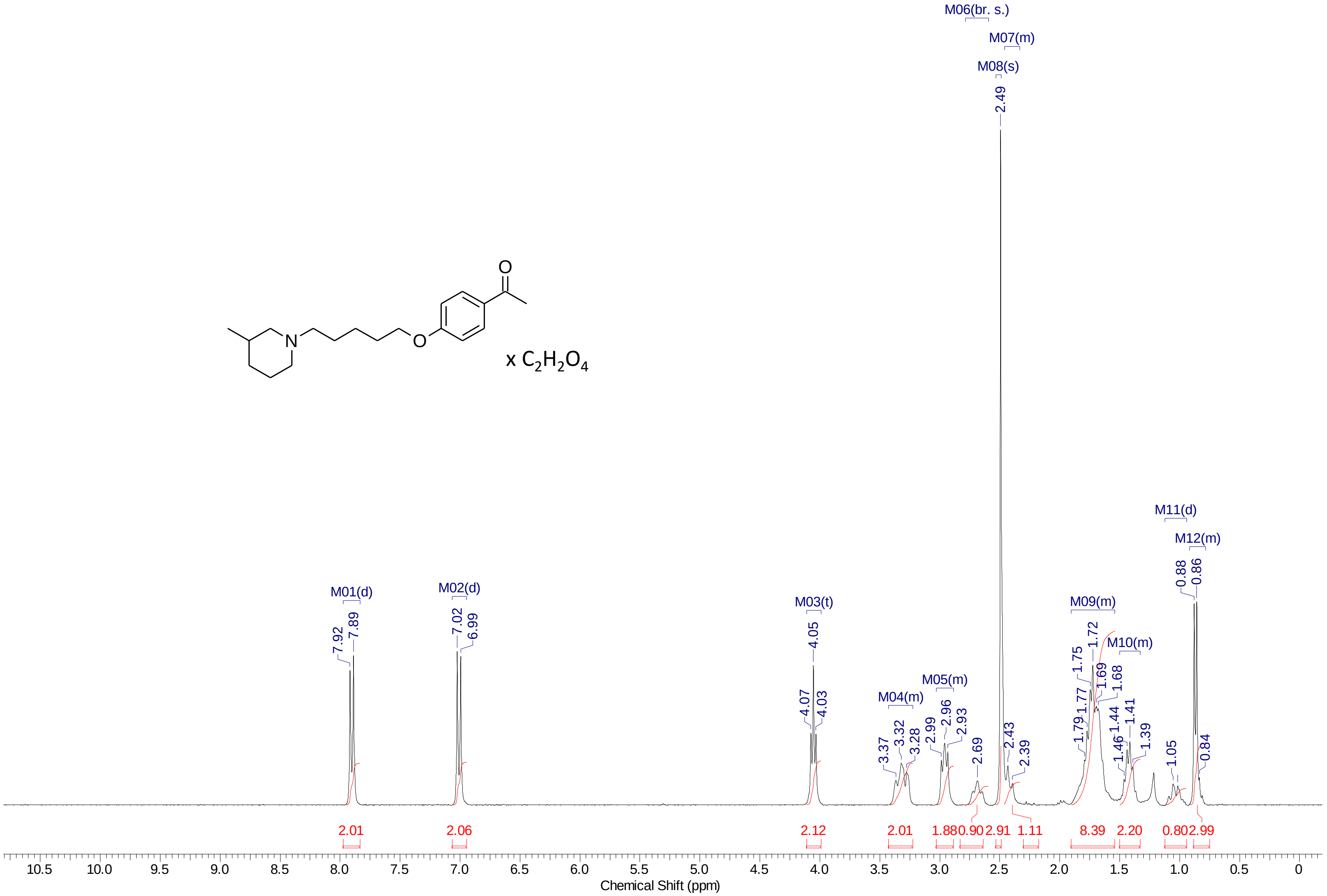

Figure S9. ^{1}H NMR spectrum 1-(4-((5-(3-methylpiperidin-1-yl)pentyl)oxy)phenyl)ethan-1-one hydrogen oxalate (**6**)

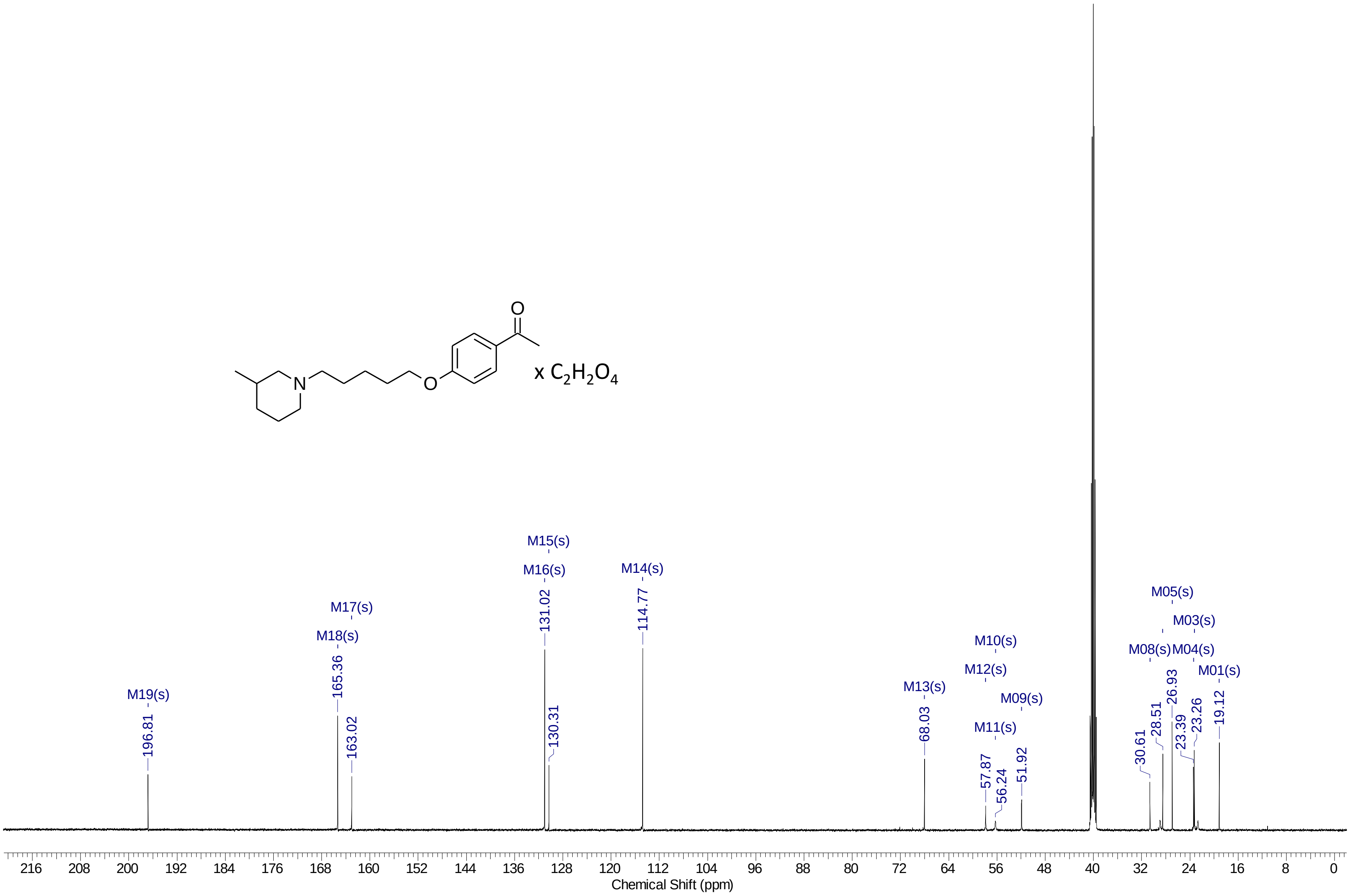

Figure S10. ^{13}C NMR spectrum of 1-(4-((5-(3-methylpiperidin-1-yl)pentyl)oxy)phenyl)ethan-1-one hydrogen oxalate (**6**)

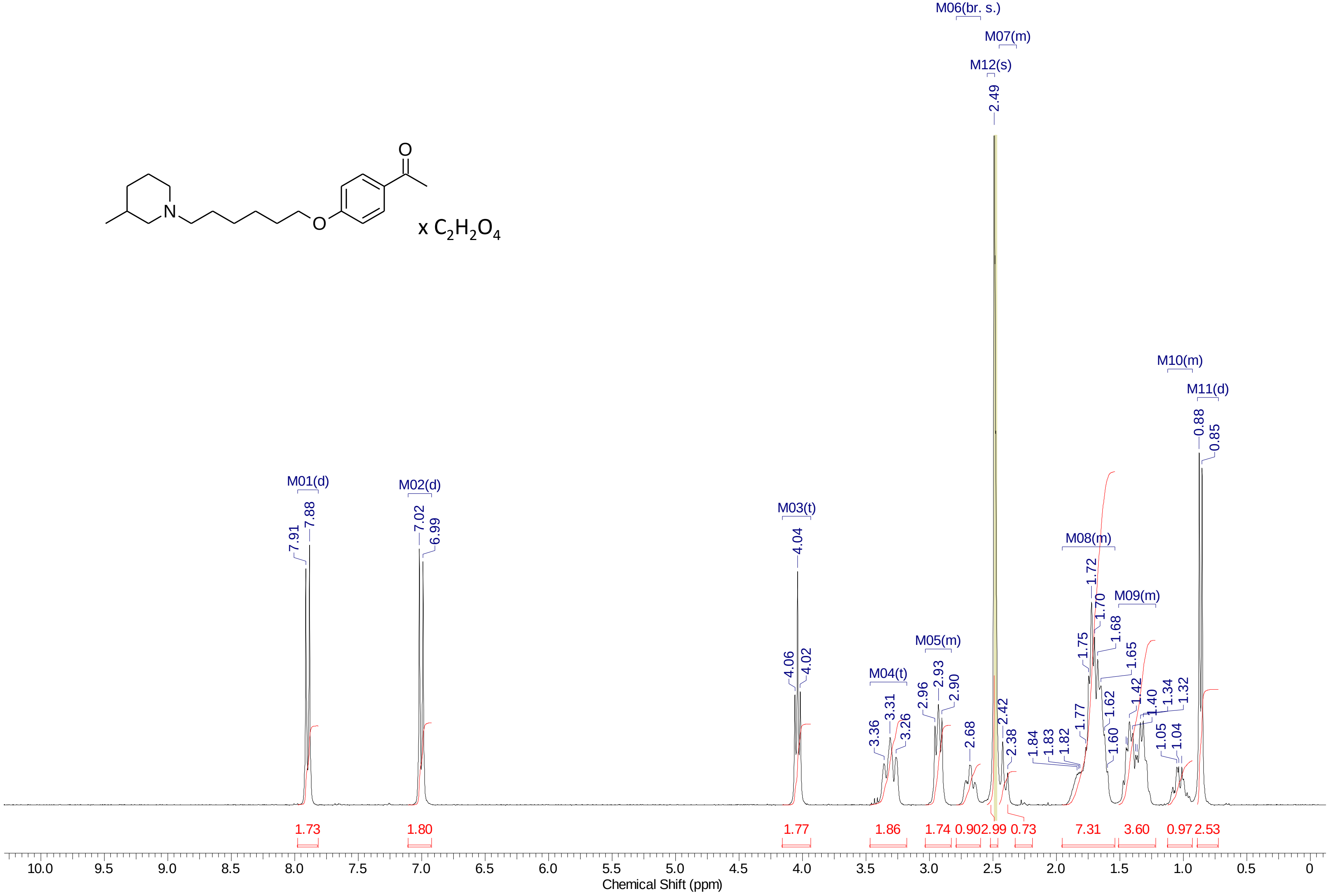

Figure S11. ^{1}H NMR spectrum of 1-(4-((6-(3-methylpiperidin-1-yl)hexyl)oxy)phenyl)ethan-1-one hydrogen oxalate (**7**)

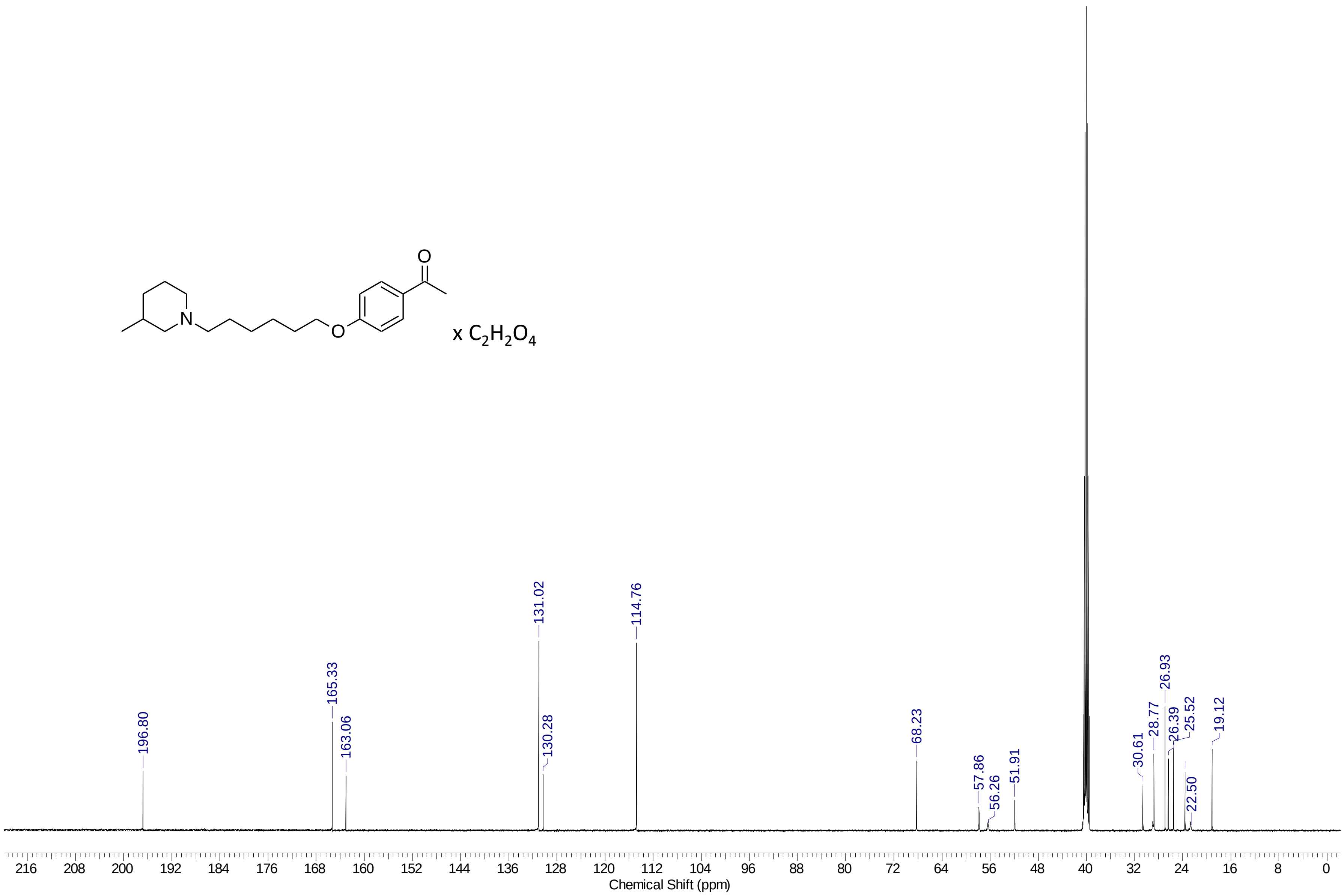

Figure S12. ^{13}C NMR spectrum of 1-(4-((6-(3-methylpiperidin-1-yl)hexyl)oxy)phenyl)ethan-1-one hydrogen oxalate (**7**)

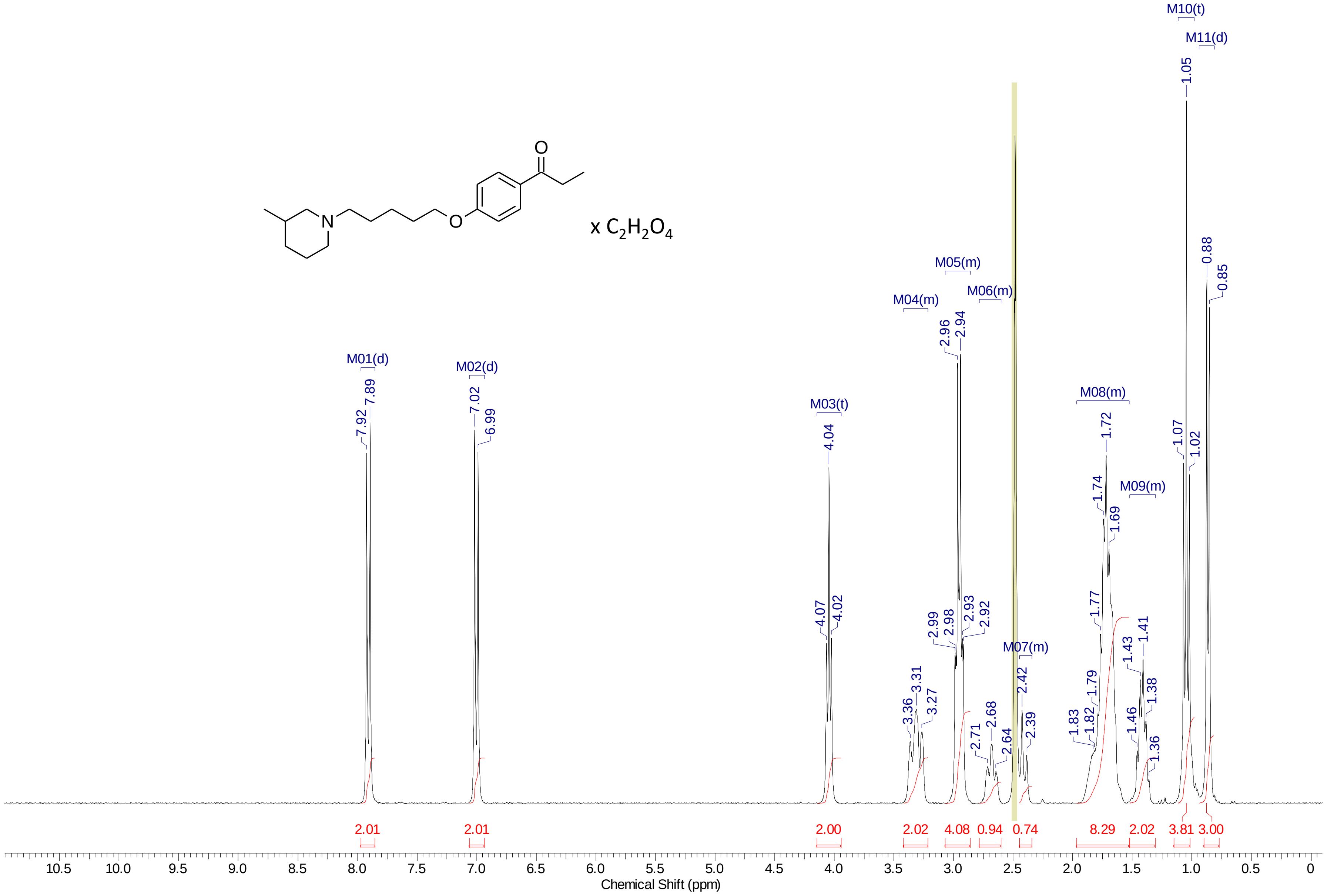

Figure S13. ^{1}H NMR spectrum of 1-(4-((5-(3-methylpiperidin-1-yl)pentyl)oxy)phenyl)propan-1-one hydrogen oxalate (**8**)

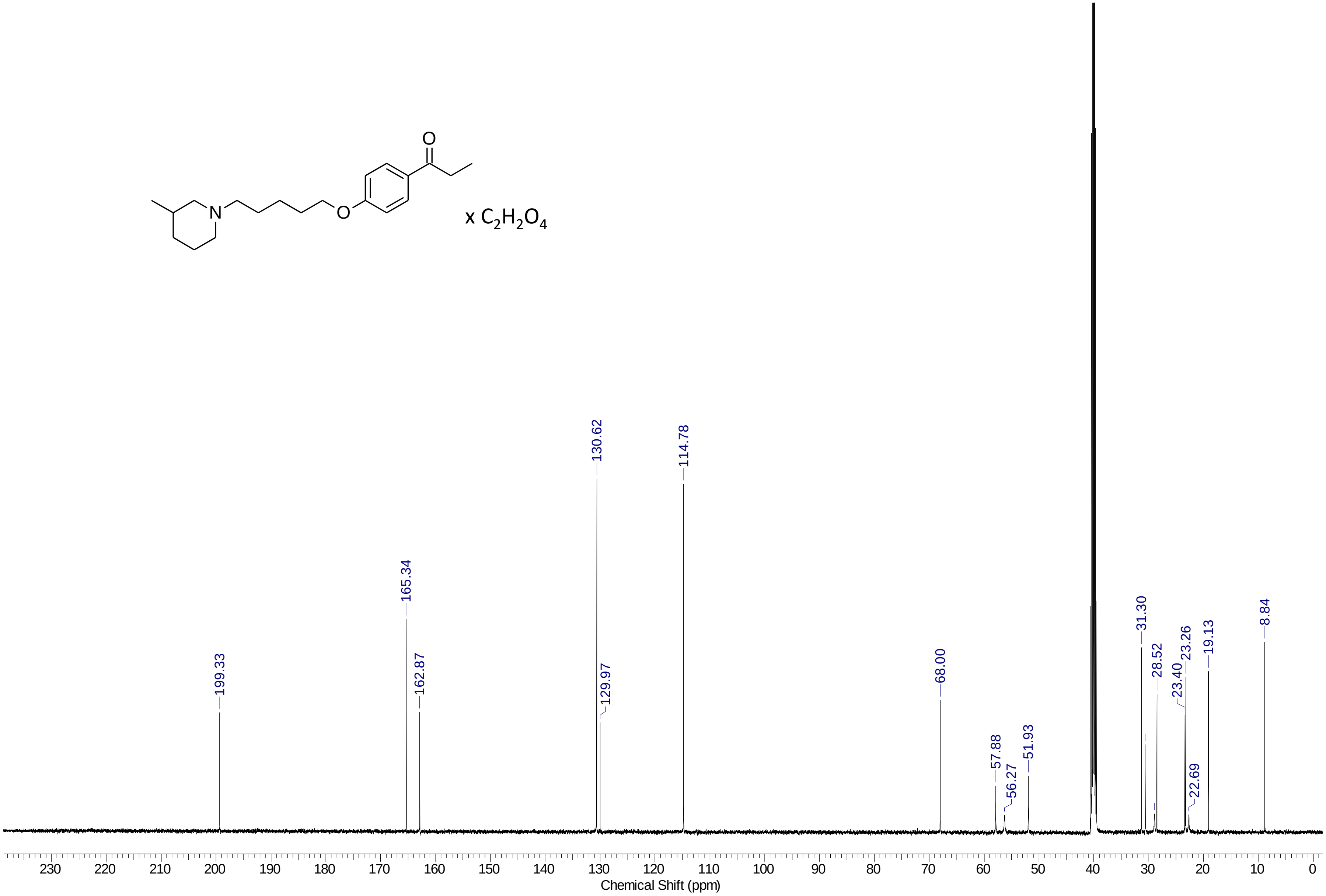

Figure S14. ^{13}C NMR spectrum of 1-(4-((5-(3-methylpiperidin-1-yl)pentyl)oxy)phenyl)propan-1-one hydrogen oxalate (**8**)

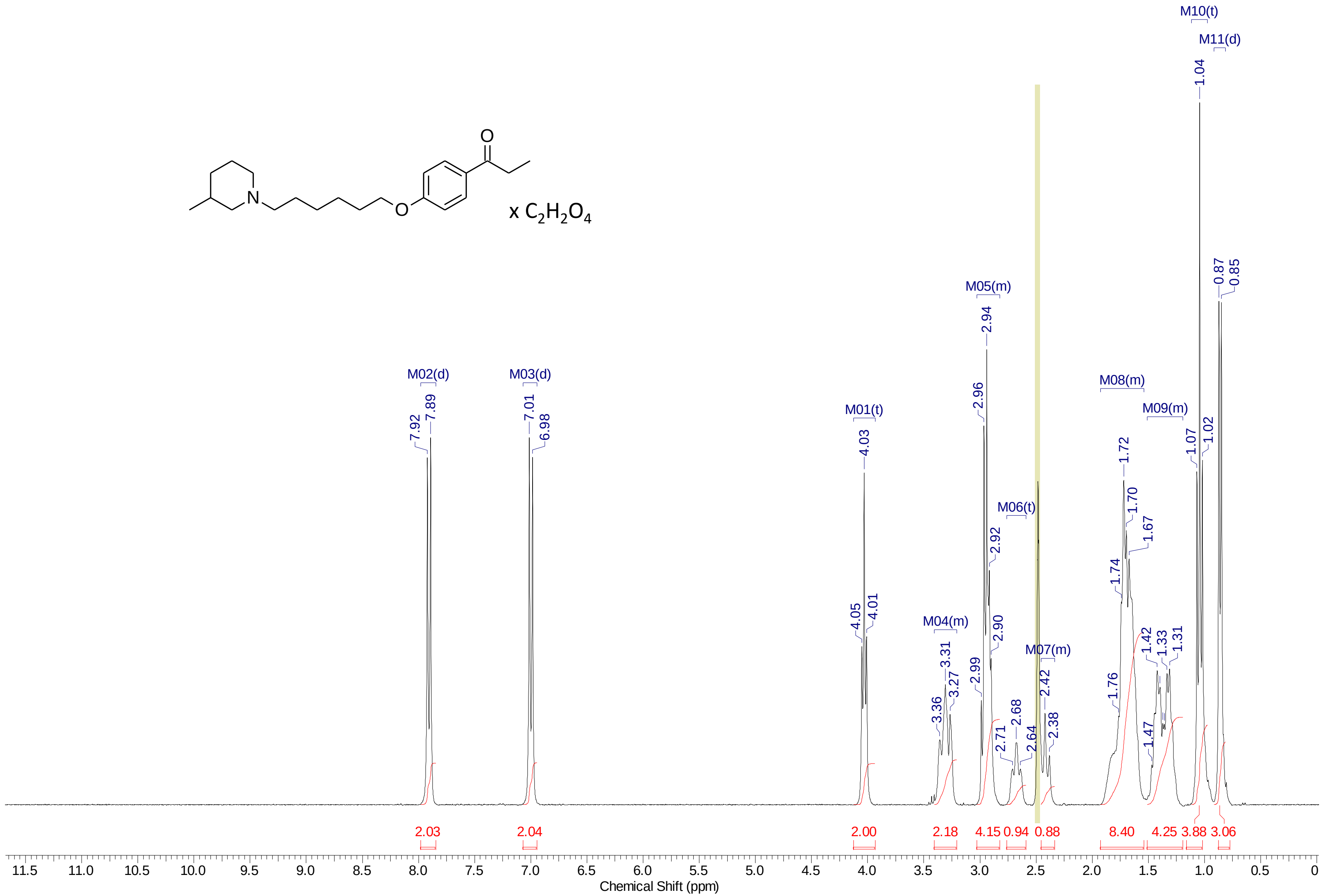

Figure S15. ^{1}H NMR spectrum of 1-(4-((5-(3-methylpiperidin-1-yl)pentyl)oxy)phenyl)propan-1-one hydrogen oxalate (**9**)

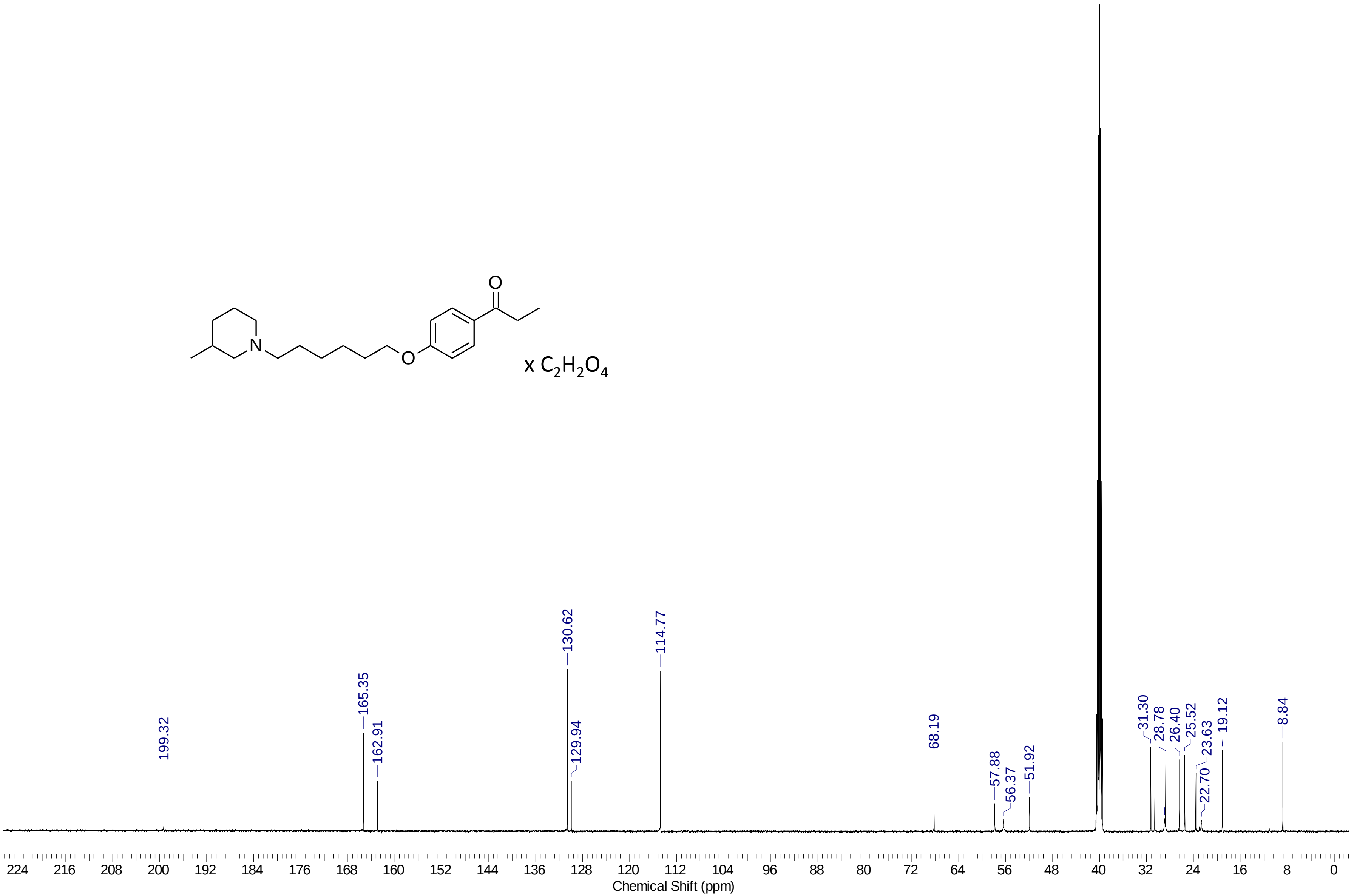

Figure S16. ^{13}C NMR spectrum of 1-(4-((5-(3-methylpiperidin-1-yl)pentyl)oxy)phenyl)propan-1-one hydrogen oxalate (**9**)

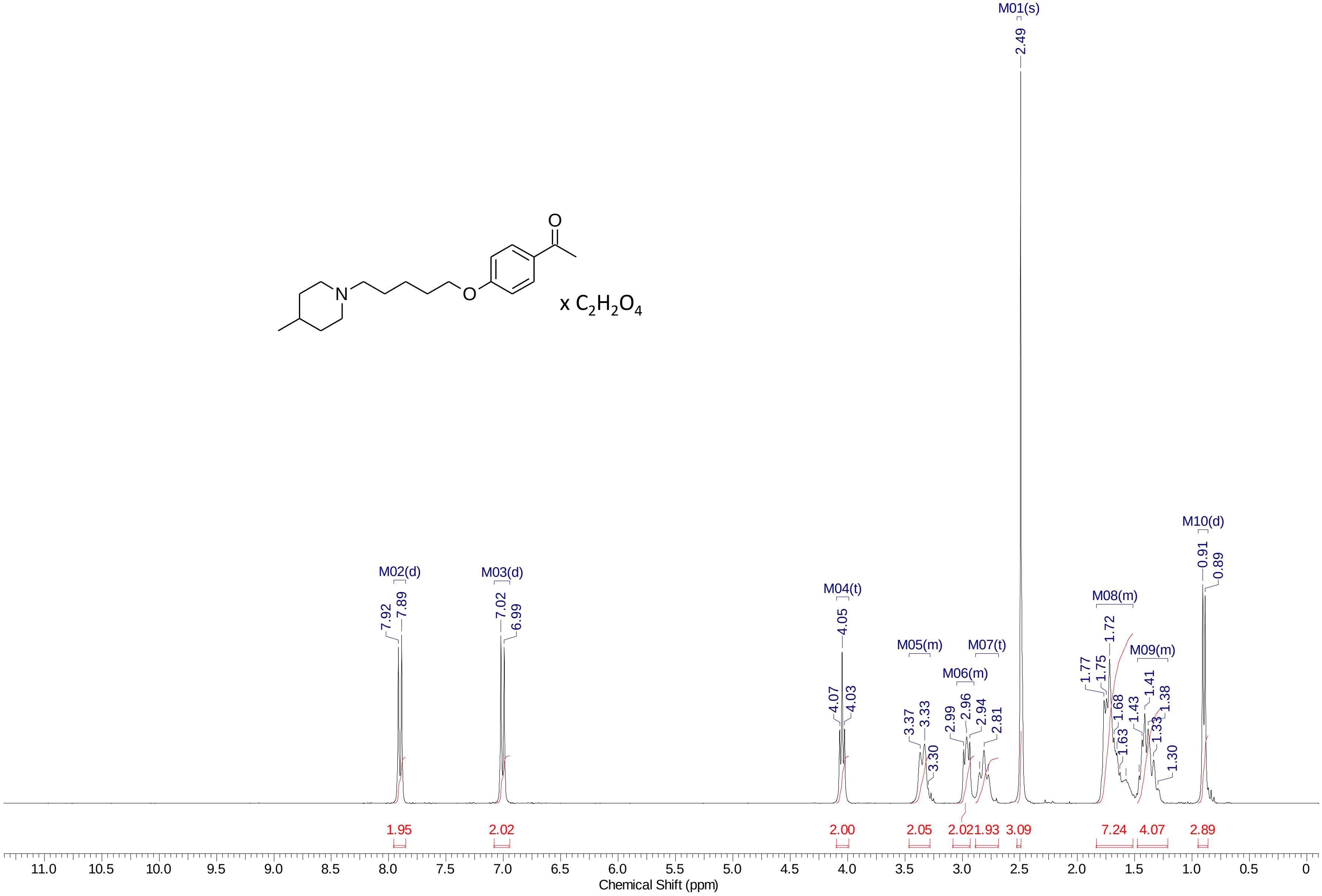

Figure S17. ^{1}H NMR spectrum of 1-(4-((5-(4-methylpiperidin-1-yl)pentyl)oxy)phenyl)ethan-1-one hydrogen oxalate (**10**)

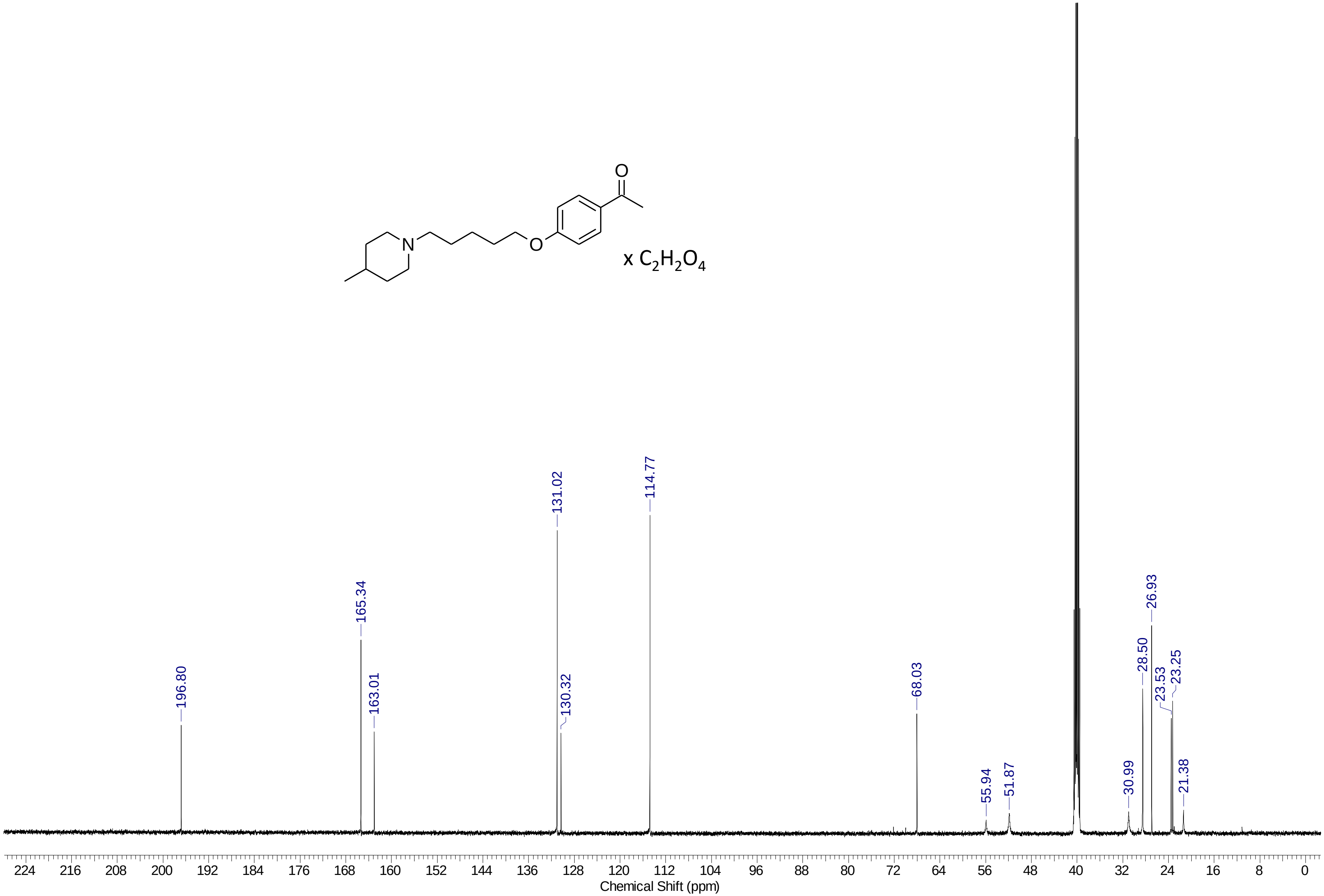

Figure S18. ^{13}C NMR spectrum of 1-(4-((5-(4-methylpiperidin-1-yl)pentyl)oxy)phenyl)ethan-1-one hydrogen oxalate (**10**)

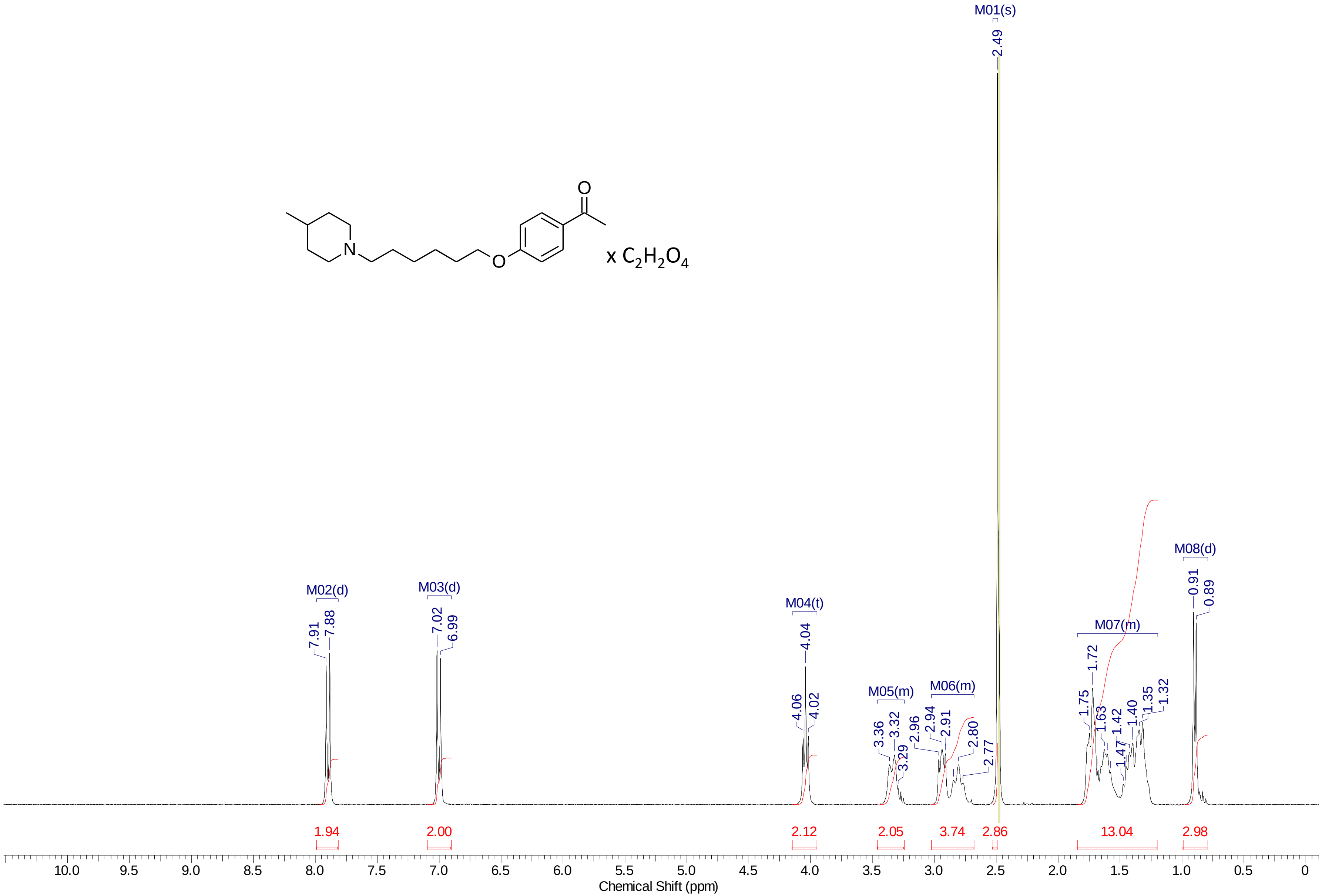

Figure S19. ^{1}H NMR spectrum of 1-(4-((6-(4-methylpiperidin-1-yl)hexyl)oxy)phenyl)ethan-1-one hydrogen oxalate (**11**)

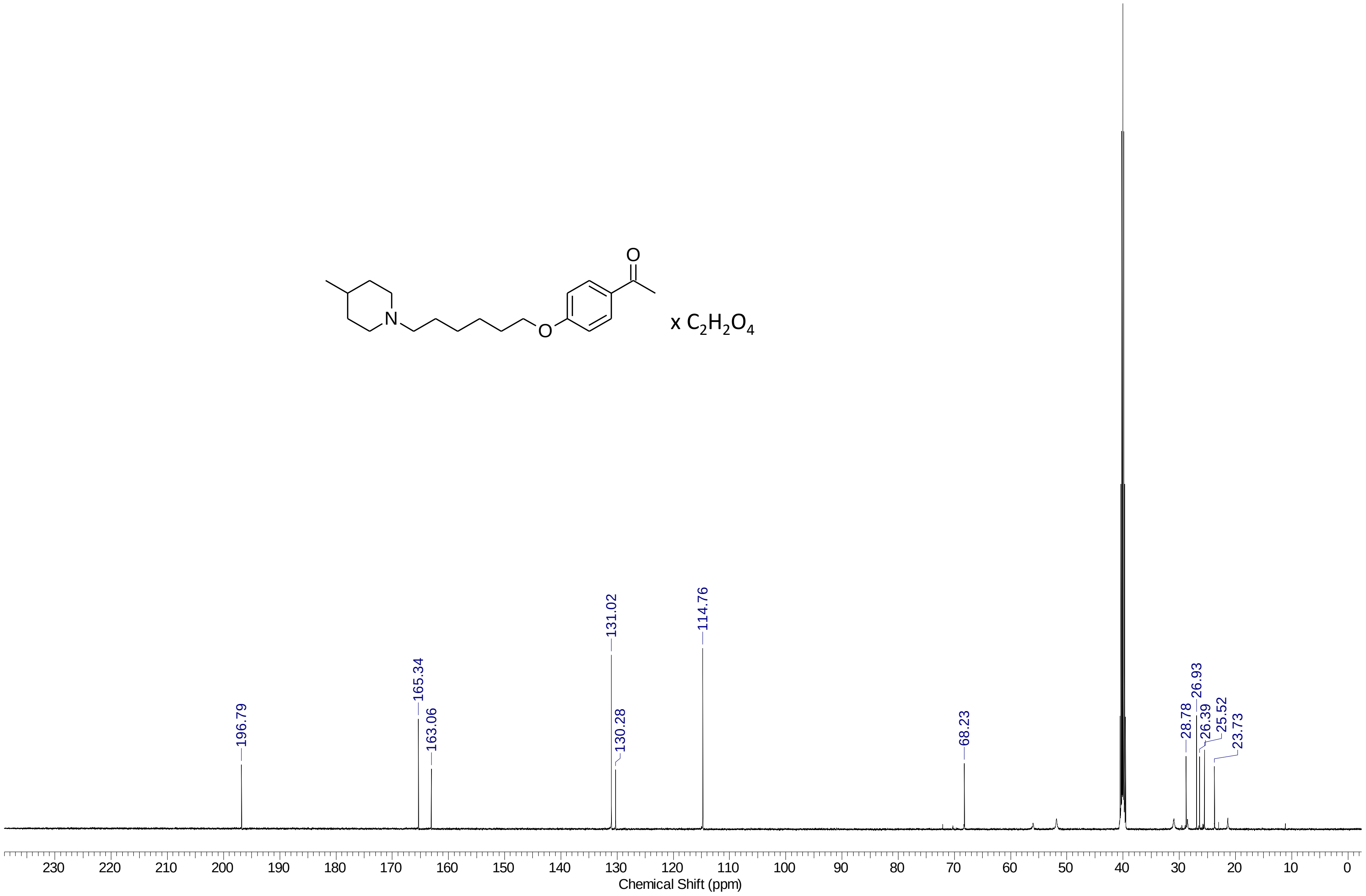

Figure S20. ^{13}C NMR spectrum of 1-(4-((6-(4-methylpiperidin-1-yl)hexyl)oxy)phenyl)ethan-1-one hydrogen oxalate (**11**)

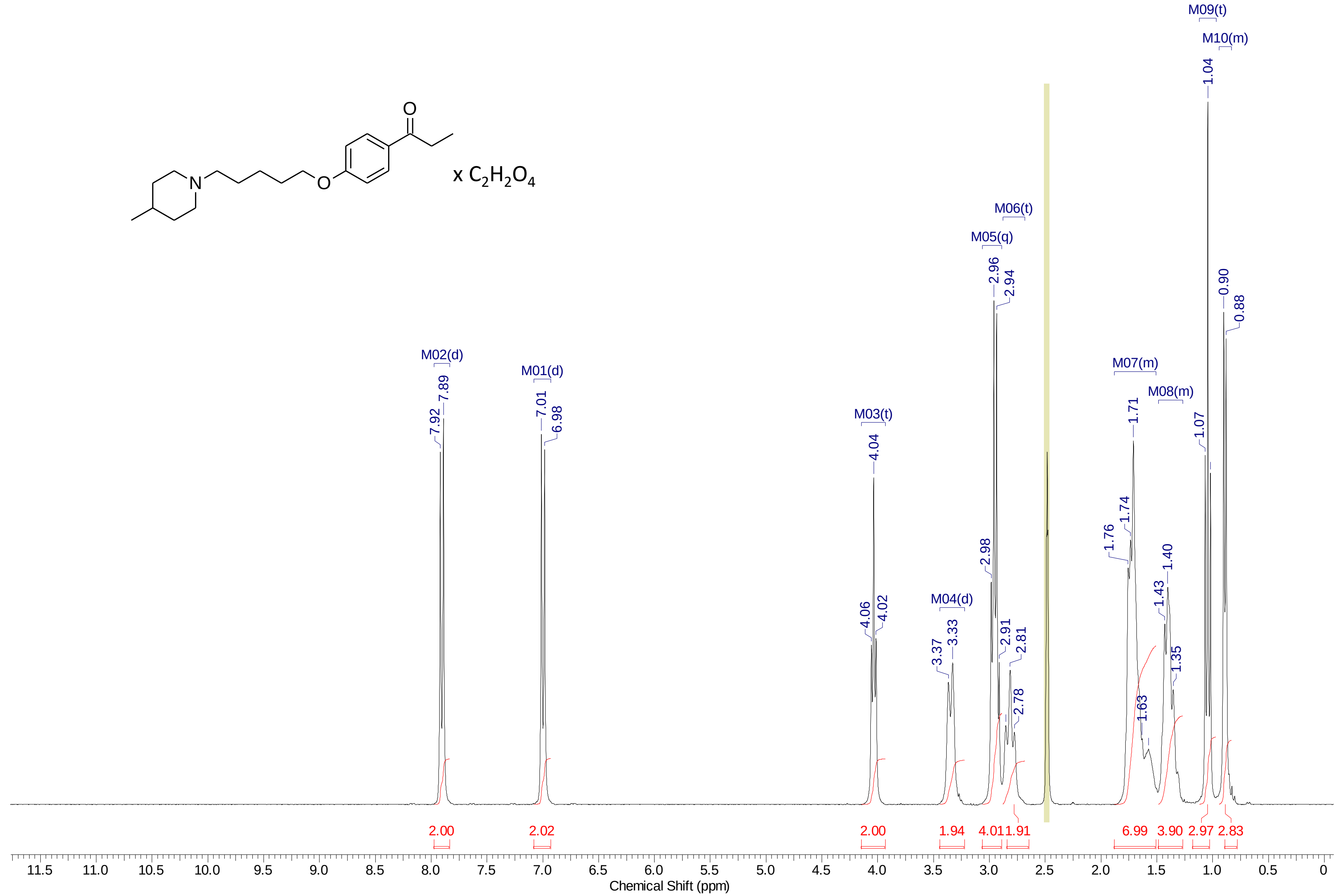

Figure S21. ^{1}H NMR spectrum of 1-(4-((5-(4-methylpiperidin-1-yl)pentyl)oxy)phenyl)propan-1-one hydrogen oxalate (**12**)

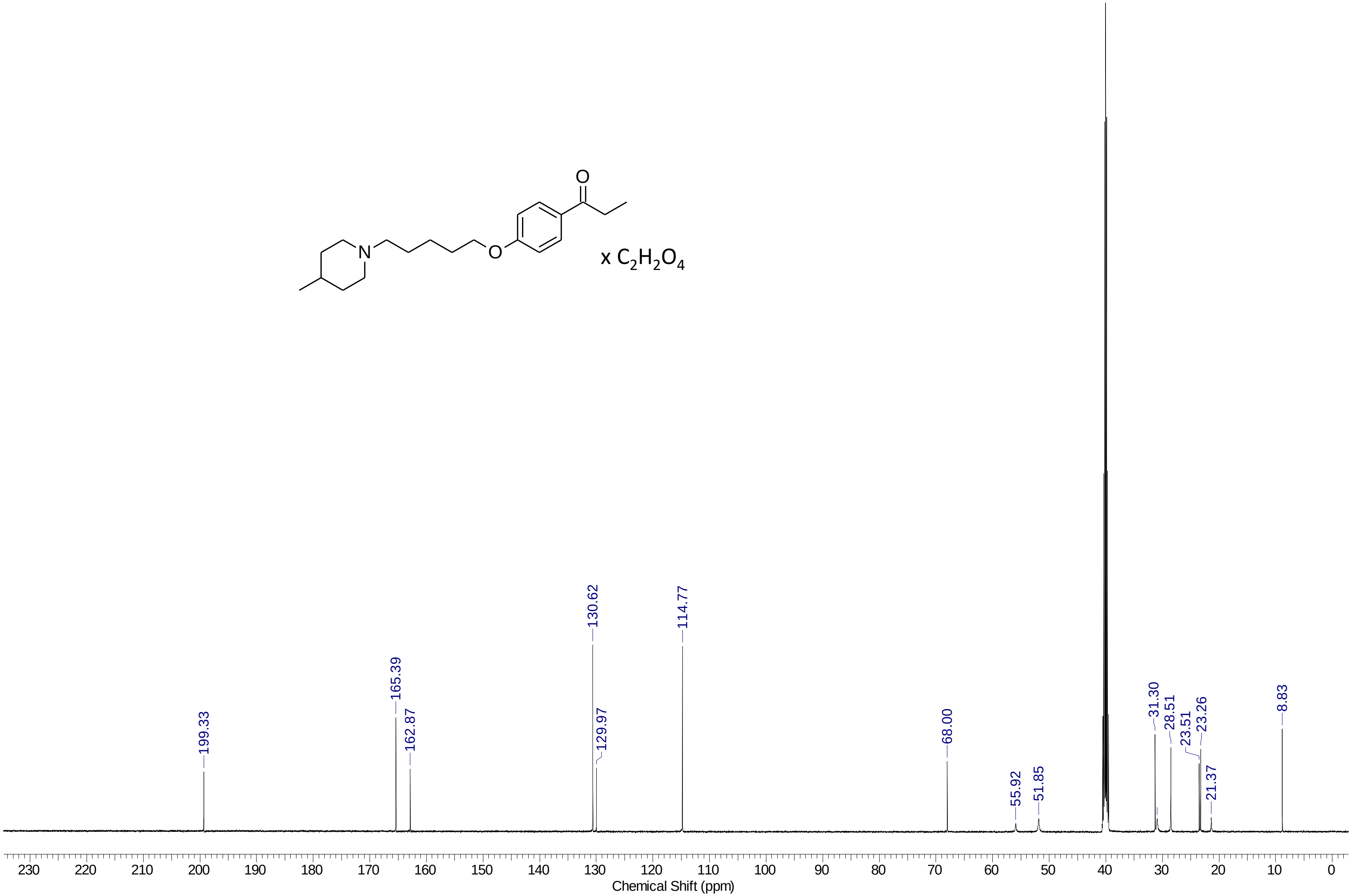

Figure S22. ^{13}C NMR spectrum of 1-(4-((5-(4-methylpiperidin-1-yl)pentyl)oxy)phenyl)propan-1-one hydrogen oxalate (**12**)

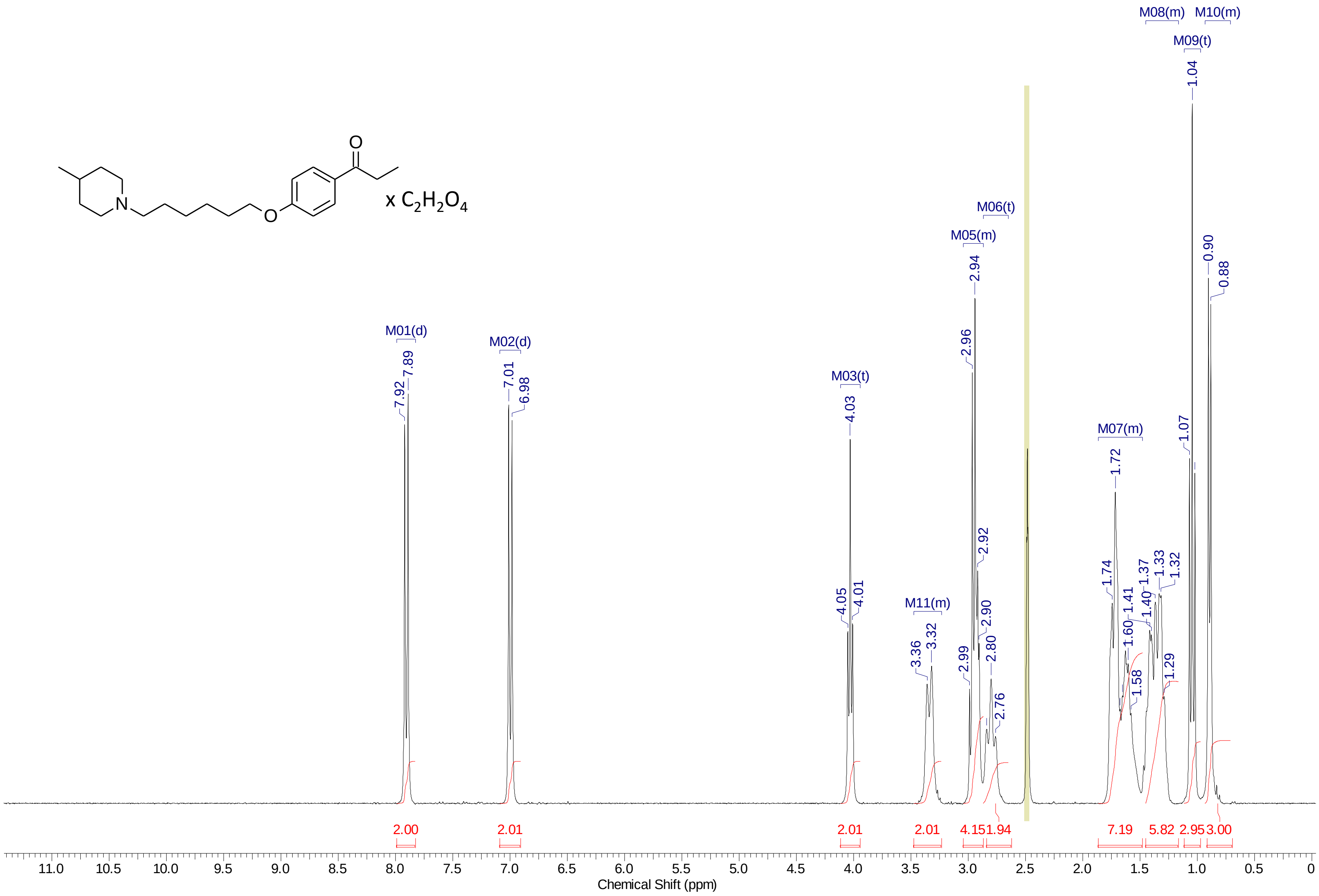

Figure S23. ^{1}H NMR spectrum of 1-(4-((6-(4-methylpiperidin-1-yl)hexyl)oxy)phenyl)propan-1-one hydrogen oxalate (**13**)

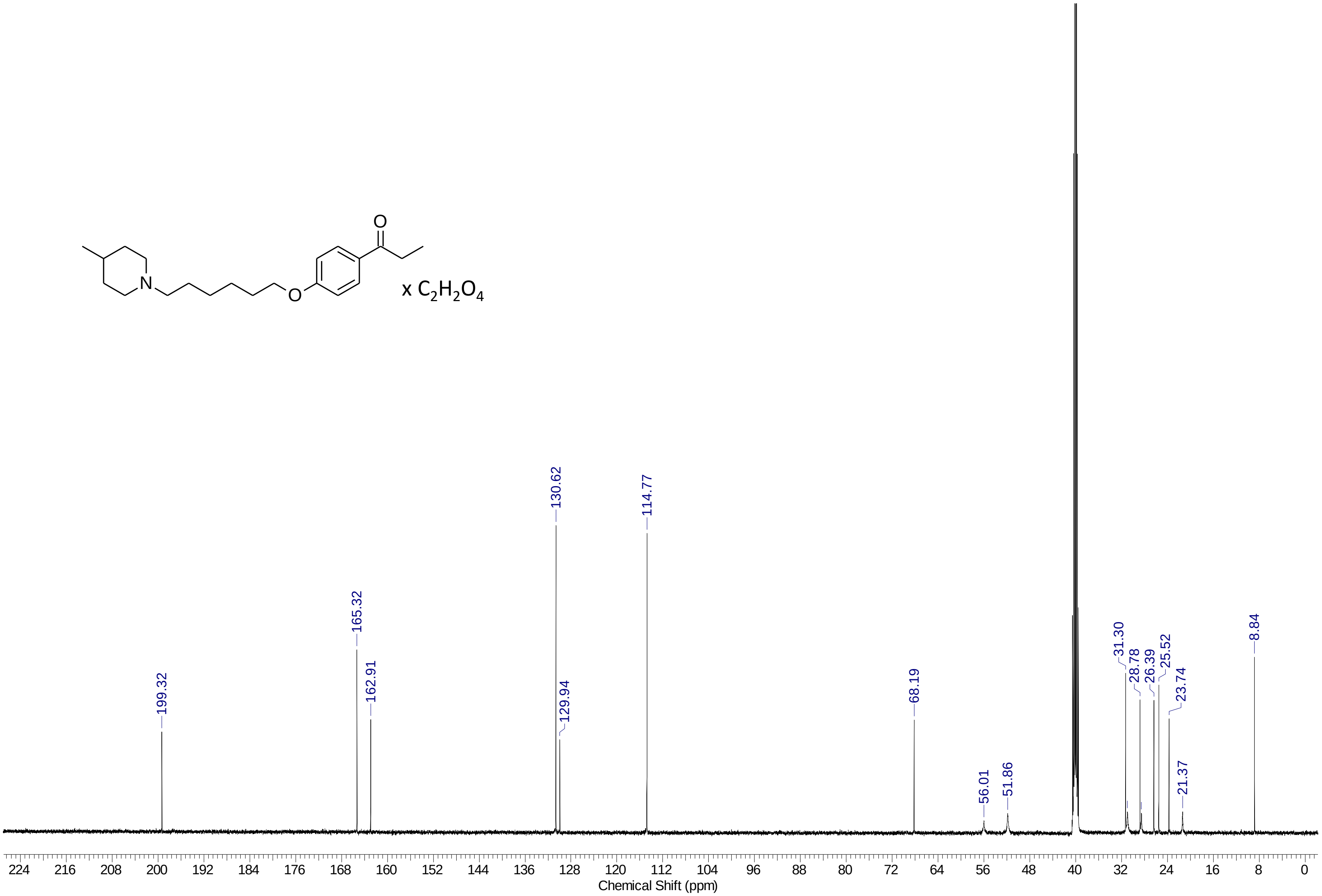

Figure S24. ^{13}C NMR spectrum of 1-(4-((6-(4-methylpiperidin-1-yl)hexyl)oxy)phenyl)propan-1-one hydrogen oxalate (**13**)

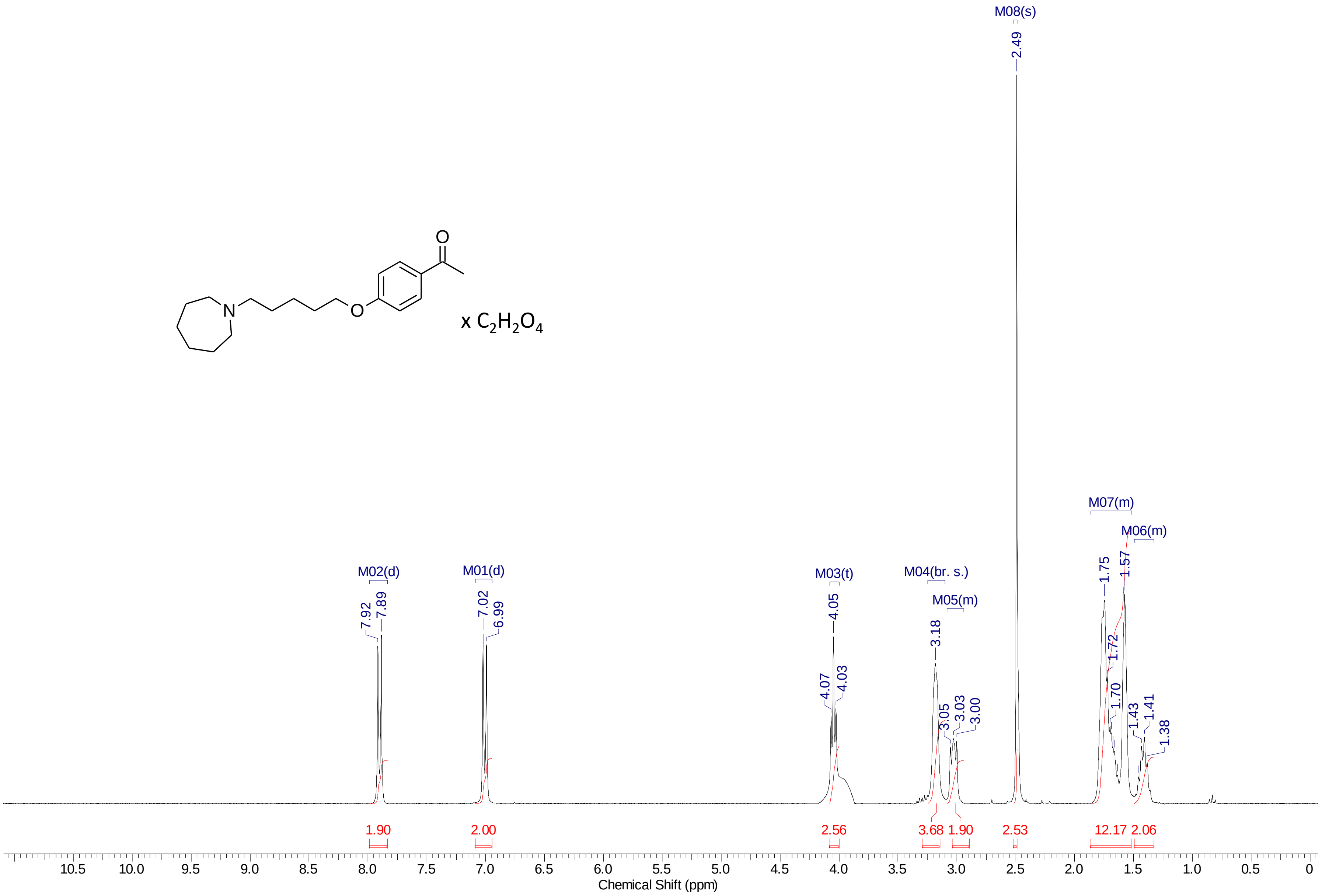

Figure S25. ^{1}H NMR spectrum of 1-(4-((5-(azepan-1-yl)pentyl)oxy)phenyl)ethan-1-one hydrogen oxalate (**14**)

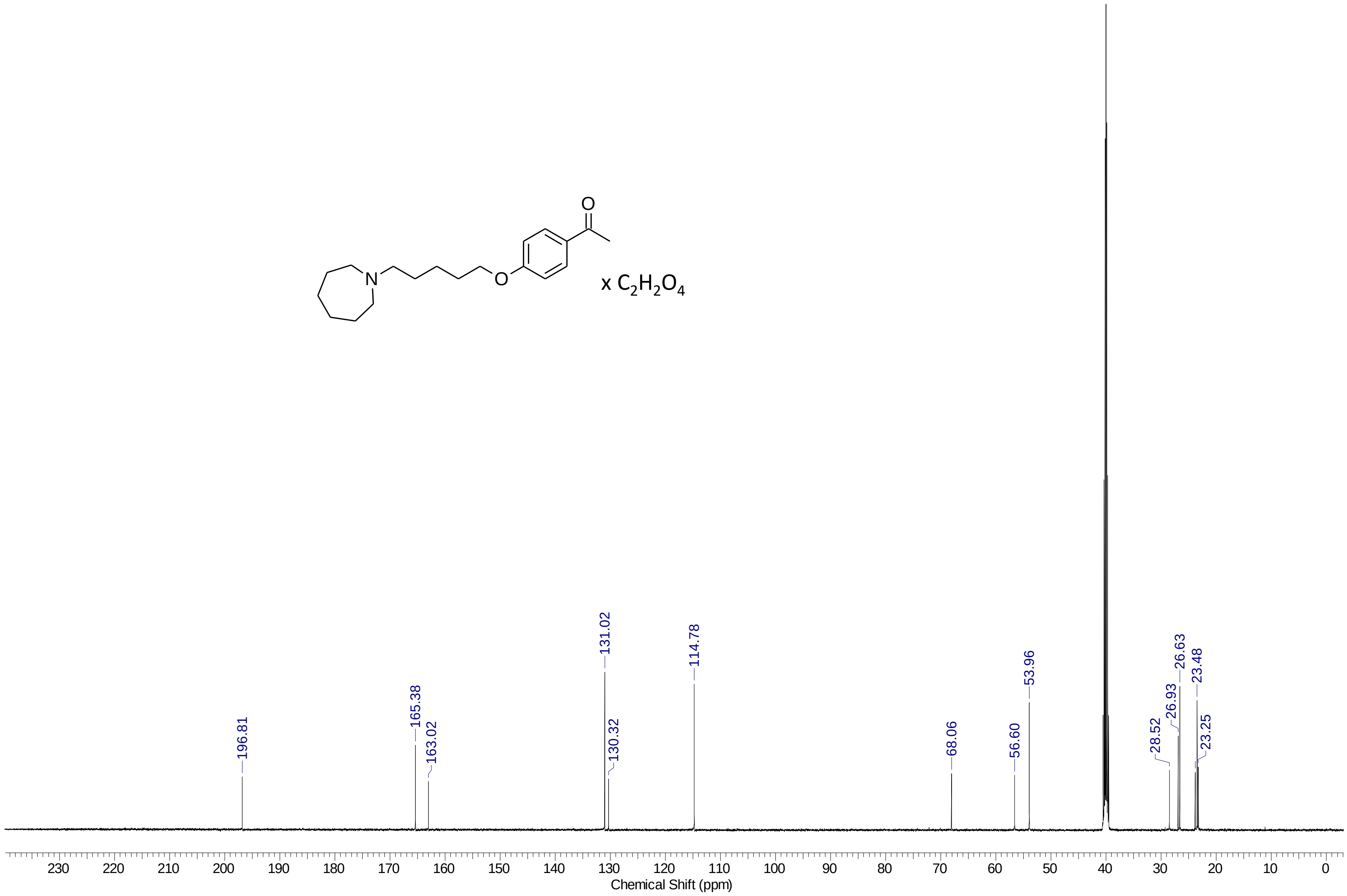

Figure S26. ^{13}C NMR spectrum of 1-(4-((5-(azepan-1-yl)pentyl)oxy)phenyl)ethan-1-one hydrogen oxalate (**14**)

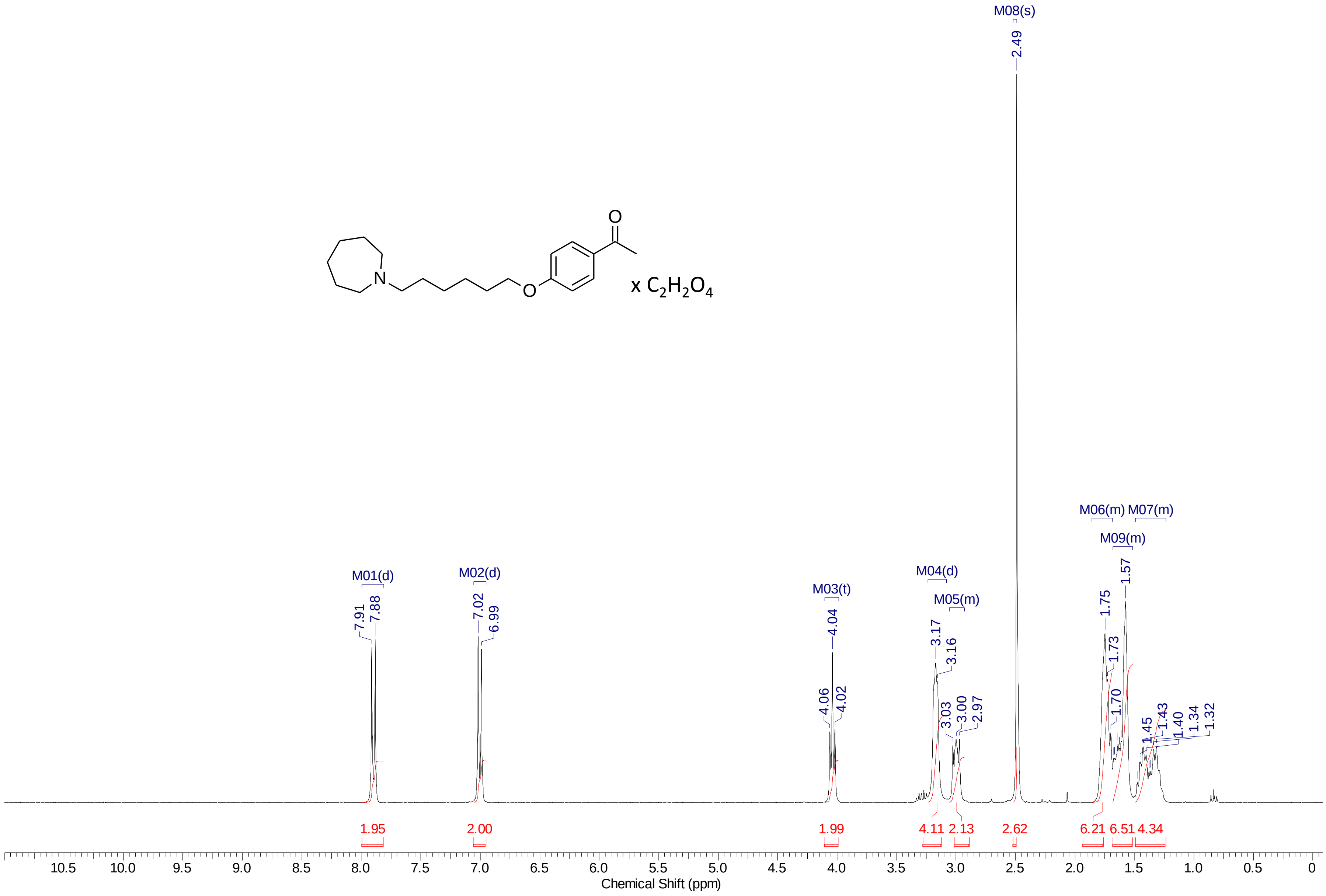

Figure S27. ^{1}H NMR spectrum of 1-(4-((5-(azepan-1-yl)hexyl)oxy)phenyl)ethan-1-one hydrogen oxalate (**15**)

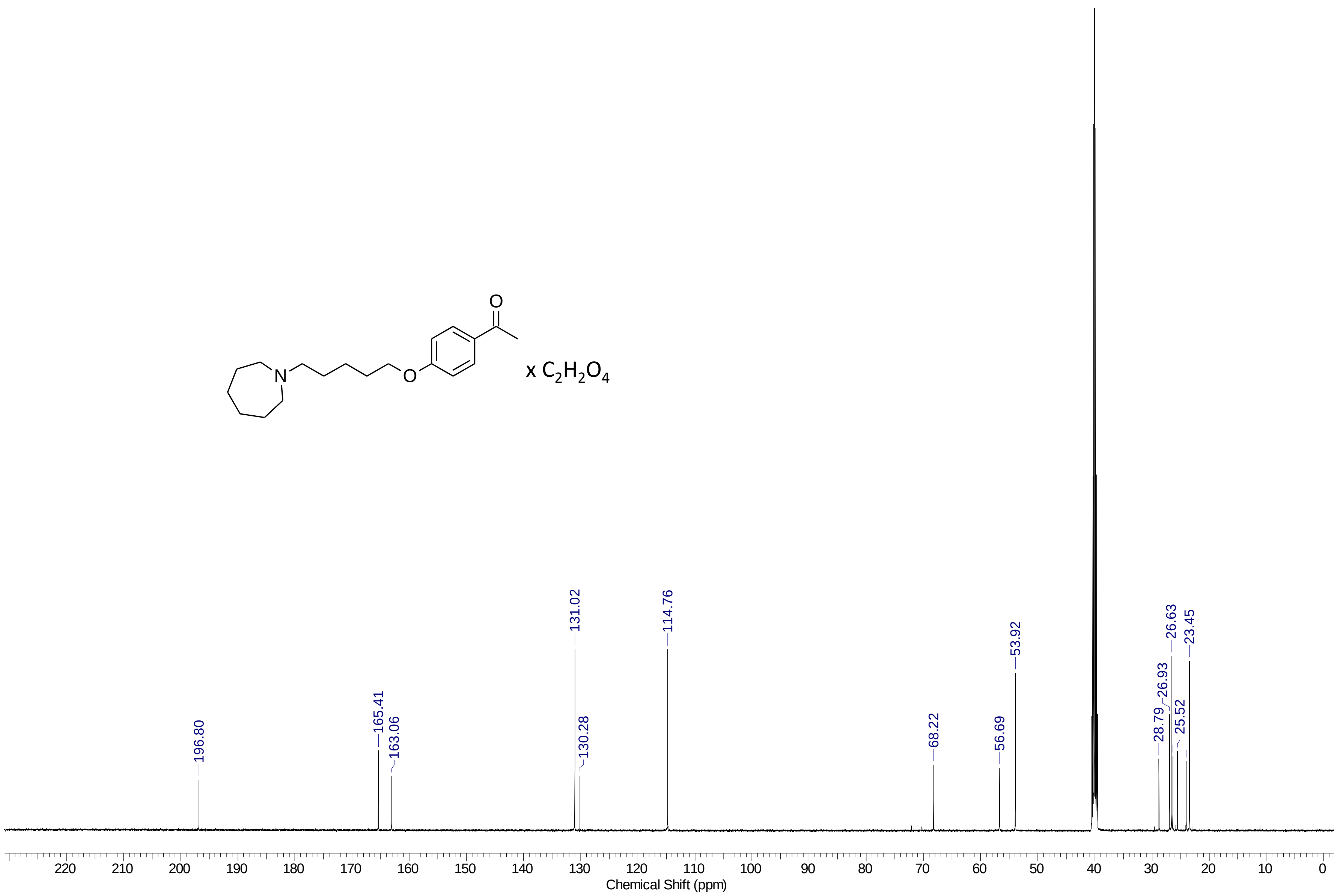

Figure S28. ^{13}C NMR spectrum of 1-(4-((5-(azepan-1-yl)hexyl)oxy)phenyl)ethan-1-one hydrogen oxalate (**15**)

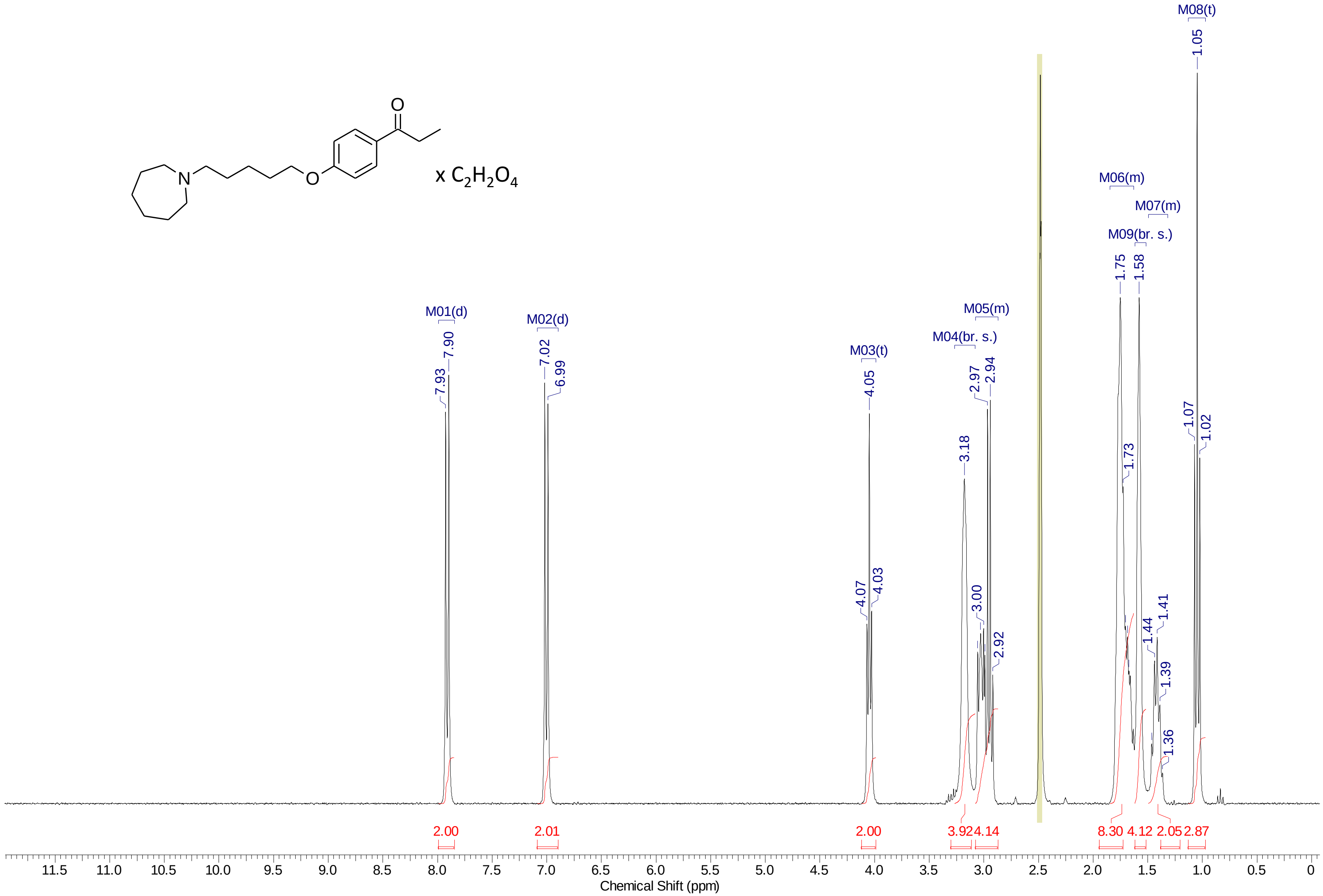

Figure S29. ^{1}H NMR spectrum of 1-(4-((5-(azepan-1-yl)pentyl)oxy)phenyl)propan-1-one hydrogen oxalate (**16**)

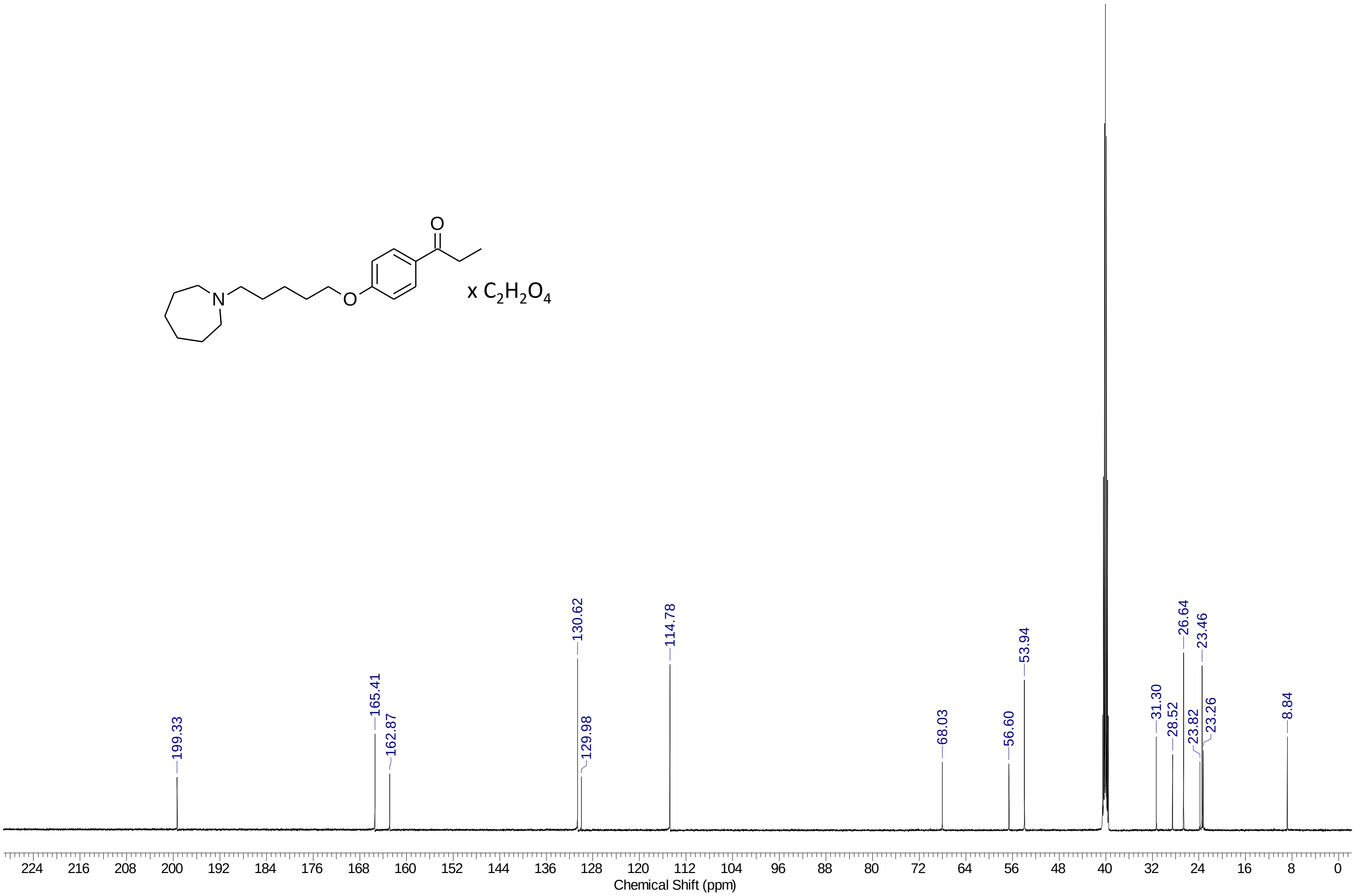

Figure S30. ^{13}C NMR spectrum of 1-(4-((5-(azepan-1-yl)pentyl)oxy)phenyl)propan-1-one hydrogen oxalate (**16**)

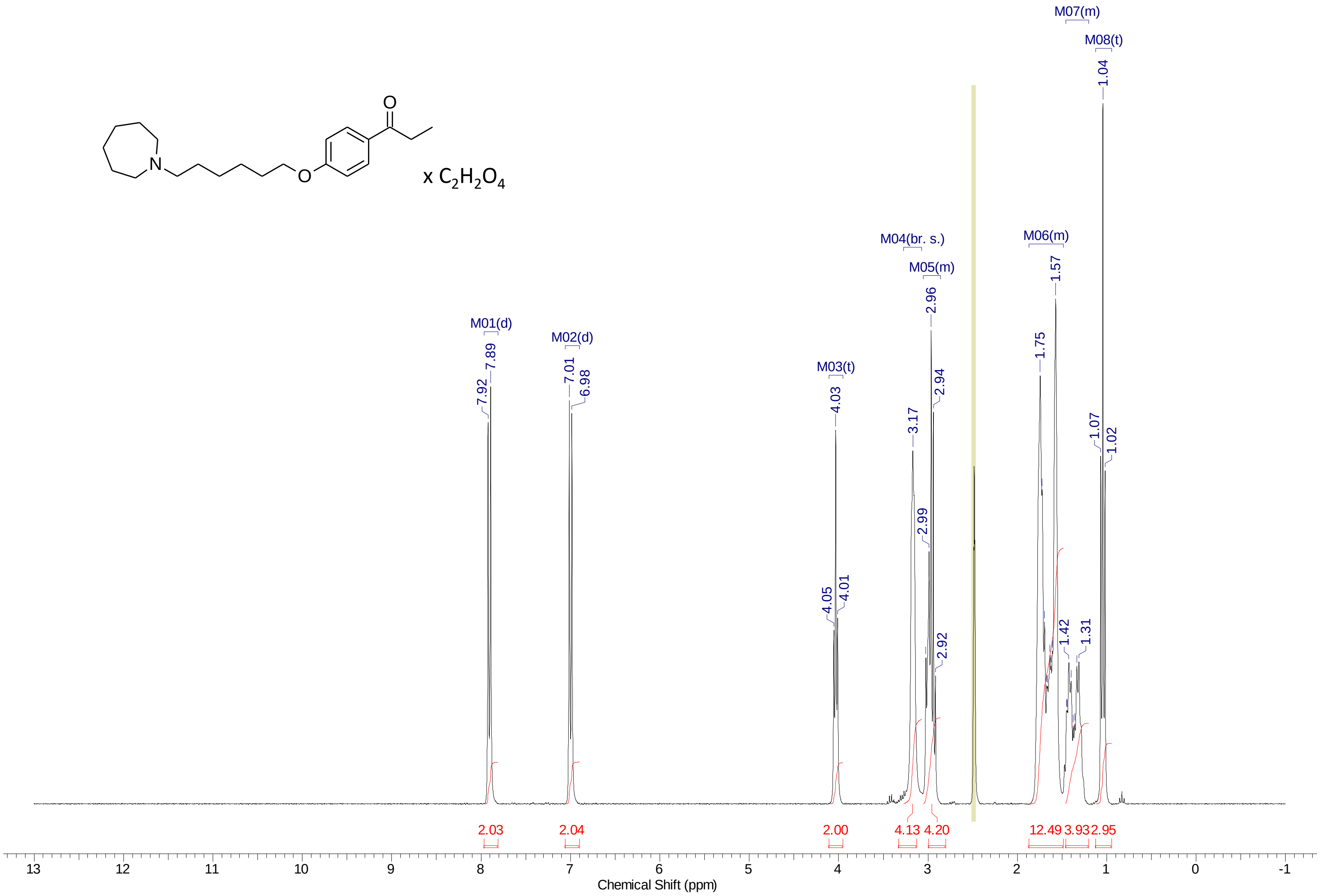

Figure S31. ^{1}H NMR spectrum of 1-(4-((6-(azepan-1-yl)hexyl)oxy)phenyl)propan-1-one hydrogen oxalate (**17**)

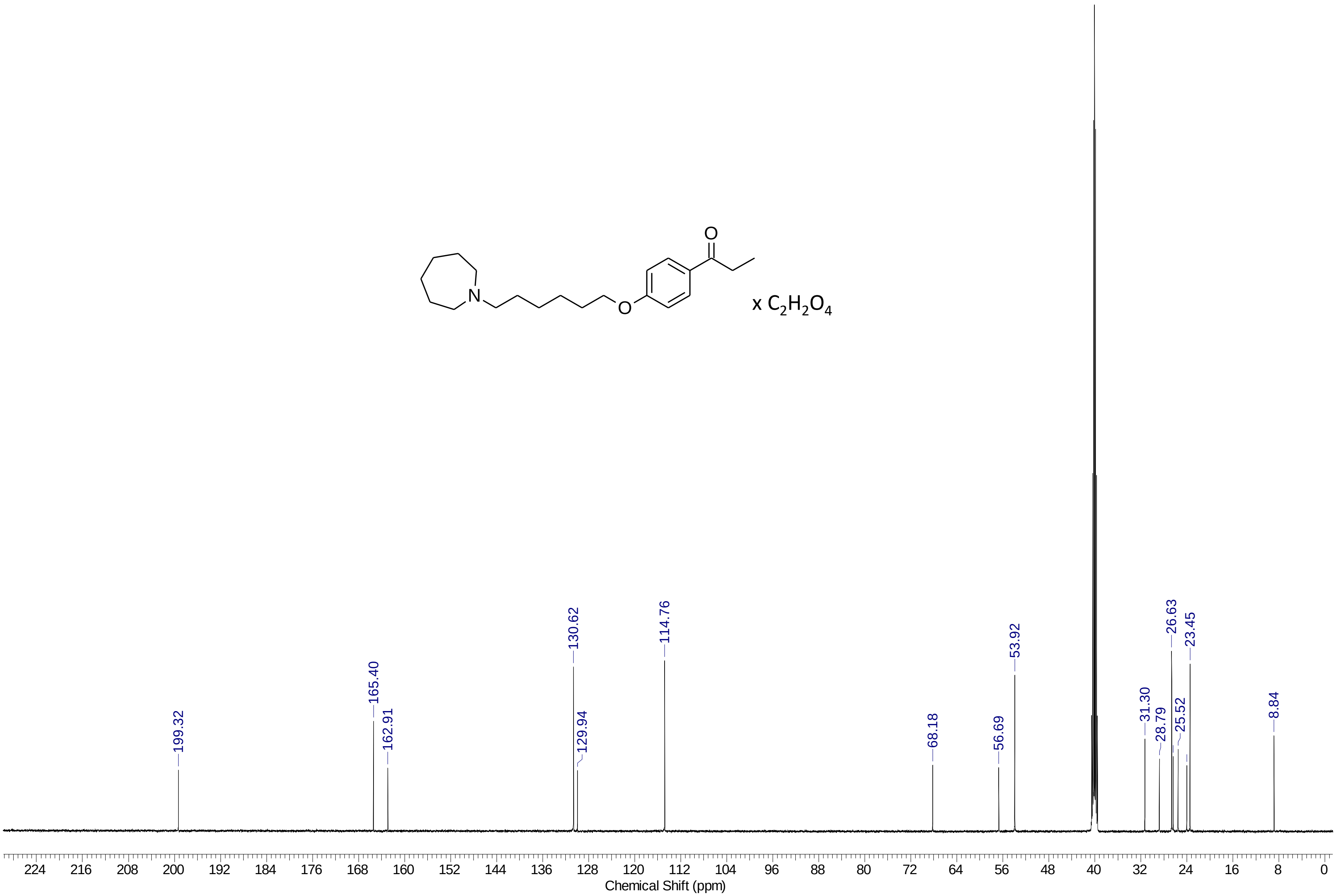

Figure S32. ^{13}C NMR spectrum of 1-(4-((6-(azepan-1-yl)hexyl)oxy)phenyl)propan-1-one hydrogen oxalate (**17**)
