# Supplementary material for: Multitargeting Histamine H3 Receptor Ligands among Acetyl- and Propionyl-Phenoxyalkyl Derivatives
Source: Molecules. 2023 Mar 3;28(5):2349. doi: 10.3390/molecules28052349 (PMC10005104; doi:10.3390/molecules28052349)
Supplement: Supplementary file 1 [file molecules-28-02349-s001.zip › Preliminary calculations - SwissAdme.pdf]

Preliminary calculations done using SwissAdme server (http://www.swissadme.ch/ (Last accessed on 28 December 2022)

name	Compound - structure	Molecular weight	Consensus Log P
**Lead 2**	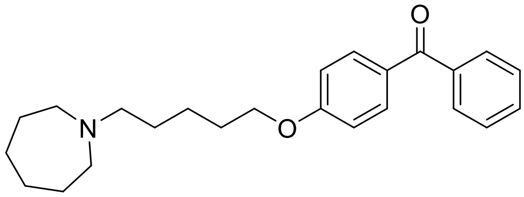	365	4.82
**15**	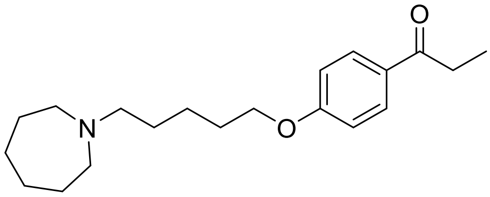	317	4.16
**14**	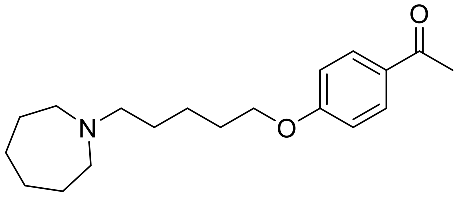	303	3.79
**Lead 3**	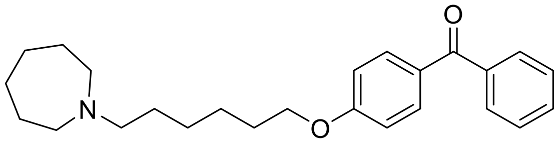	380	5.18
**17**	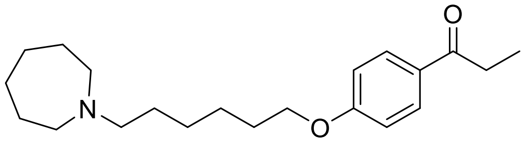	331	4.45
**16**	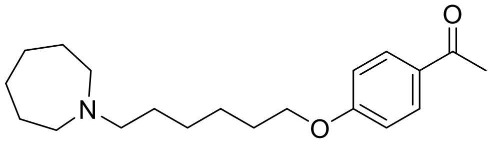	317	4.15
