# Supplementary figures and images for: Multitargeting Histamine H3 Receptor Ligands among Acetyl- and Propionyl-Phenoxyalkyl Derivatives
Source: Molecules. 2023 Mar 3;28(5):2349. doi: 10.3390/molecules28052349 (PMC10005104; doi:10.3390/molecules28052349)

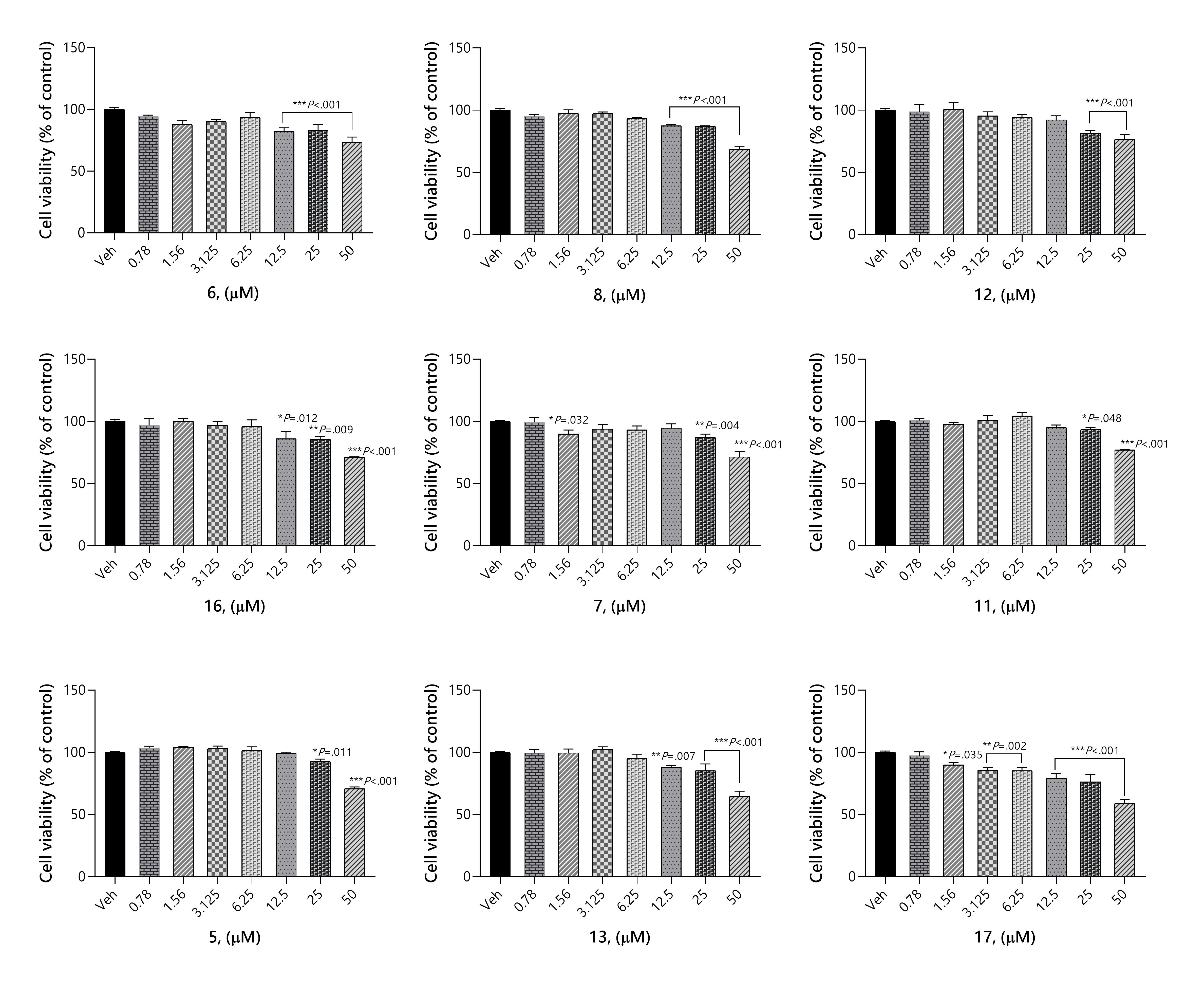

Supplement: Supplementary file 1 [file molecules-28-02349-s001.zip › Figure 1. Toxicity evaluation in HepG2 cells.jpg]

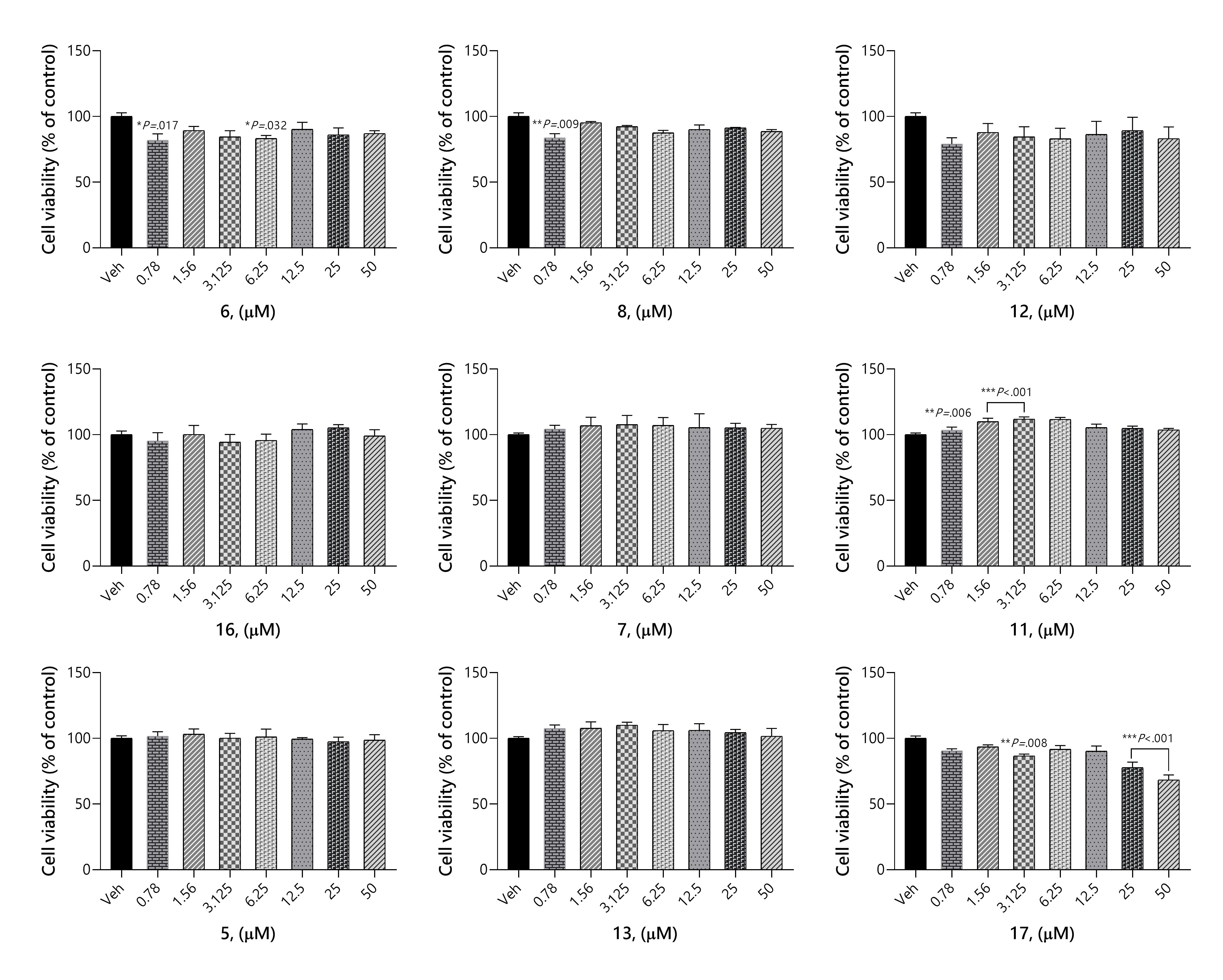

Supplement: Supplementary file 1 [file molecules-28-02349-s001.zip › Figure 2. Toxicity evaluation in SH-SY5Y cells.jpg]
